# Implications of 2D versus 3D surveys to measure the abundance and composition of benthic coral reef communities

**DOI:** 10.1007/s00338-021-02118-6

**Published:** 2021-06-16

**Authors:** Niklas A. Kornder, Jose Cappelletto, Benjamin Mueller, Margaretha J. L. Zalm, Stephanie J. Martinez, Mark J. A. Vermeij, Jef Huisman, Jasper M. de Goeij

**Affiliations:** 1 Department of Freshwater and Marine Ecology, Institute for Biodiversity and Ecosystem Dynamics, University of Amsterdam, P.O. Box 94240, 1090 GE Amsterdam, The Netherlands; 2 Maritime Robotics Laboratory, Southampton Marine and Maritime Institute, Faculty of Engineering and Physical Science, University of Southampton, Southampton, SO16 7QF UK; 3 Grupo de I+D en Mecatrónica, Universidad Simón Bolívar, Baruta, Caracas, 89000 Edo. Miranda Venezuela; 4 CARMABI Foundation, Piscaderabaai z/n, P.O. Box 2090, Willemstad, Curaçao

**Keywords:** Habitat complexity, Biomass, Standing stock, Community cover composition, Relative abundance, Sponges, Algae, Coelobites, Photogrammetry

## Abstract

A paramount challenge in coral reef ecology is to estimate the abundance and composition of the communities residing in such complex ecosystems. Traditional 2D projected surface cover estimates neglect the 3D structure of reefs and reef organisms, overlook communities residing in cryptic reef habitats (e.g., overhangs, cavities), and thus may fail to represent biomass estimates needed to assess trophic ecology and reef function. Here, we surveyed the 3D surface cover, biovolume, and biomass (i.e., ash-free dry weight) of all major benthic taxa on 12 coral reef stations on the island of Curaçao (Southern Caribbean) using structure-from-motion photogrammetry, coral point counts, in situ measurements, and elemental analysis. We then compared our 3D benthic community estimates to corresponding estimates of traditional 2D projected surface cover to explore the differences in benthic community composition using different metrics. Overall, 2D cover was dominated (52 ± 2%, mean ± SE) by non-calcifying phototrophs (macroalgae, turf algae, benthic cyanobacterial mats), but their contribution to total reef biomass was minor (3.2 ± 0.6%). In contrast, coral cover (32 ± 2%) more closely resembled coral biomass (27 ± 6%). The relative contribution of erect organisms, such as gorgonians and massive sponges, to 2D cover was twofold and 11-fold lower, respectively, than their contribution to reef biomass. Cryptic surface area (3.3 ± 0.2 m^2^ m^−2^_planar reef_) comprised half of the total reef substrate, rendering two thirds of coralline algae and almost all encrusting sponges (99.8%) undetected in traditional assessments. Yet, encrusting sponges dominated reef biomass (35 ± 18%). Based on our quantification of exposed and cryptic reef communities using different metrics, we suggest adjustments to current monitoring approaches and highlight ramifications for evaluating the ecological contributions of different taxa to overall reef function. To this end, our metric conversions can complement other benthic assessments to generate non-invasive estimates of the biovolume, biomass, and elemental composition (i.e., standing stocks of organic carbon and nitrogen) of Caribbean coral reef communities.

**Supplementary Information:**

The online version contains supplementary material available at 10.1007/s00338-021-02118-6.

## Introduction

Ecological models offer insights into complex community dynamics and biogeochemical cycling within ecosystems, but depend on accurate abundance estimates (i.e., composition and biomass) of taxa comprising communities (Diaz and Rützler 2001; Van Oevelen et al. 2006). In coral reef ecology, the abundance of organisms is traditionally assessed as the percentage of the projected reef substrate covered by each organismal group (Kohler and Gill 2006; Sandin et al. 2008b). While this two-dimensional (2D) approach can be useful to produce relatively fast estimates of the ‘health’ of a reef ecosystem (e.g., coral or macroalgal cover), it ignores the complex, three-dimensional (3D) morphology of coral reefs and the reef organisms themselves (Goatley and Bellwood 2011). Planar, projected images ignore differences in volume and biomass of erect versus non-erect organisms (e.g., gorgonians versus crustose coralline algae) and do not capture the abundance and composition of hidden benthic taxa occurring in the cryptic reef habitat (e.g., holes, overhangs, crevices, cavities), also referred to as ‘coelobites’ (Choi 1982). A lack of reef-wide biomass assessments that adequately incorporate habitat complexity and cryptic habitats could therefore potentially limit our understanding of how coral reef ecosystems function at present and develop in the future.

The critical role of habitat complexity in maintaining high biodiversity and community abundance within benthic ecosystems is well established (Kostylev et al. 2005; Kovalenko et al. 2012; Tokeshi and Arakaki 2012). To assess this complexity, numerous studies recently applied structure-from-motion photogrammetry—a technique in which 3D models are generated from a stack of images depicting objects from various angles—to estimate parameters describing the 3D topographic structure of either reef surfaces (Burns et al. 2015; Leon et al. 2015; Ferrari et al. 2016; Storlazzi et al. 2016) or individual reef organisms (Figueira et al. 2015; Lavy et al. 2015; Gutierrez-Heredia et al. 2016). Structural parameters of reef surfaces and organisms were shown to be estimated with relatively high accuracy using photogrammetry in combination with underwater action cameras (Veal et al. 2010; Guo et al. 2016; but see Bryson et al. 2017) that would be small enough to access and photograph cryptic reef surfaces. While these parameters were used, for example, to better explain variation in the occurrence of mobile fish populations (Gratwicke and Speight 2005; Harborne et al. 2012), attempts to implement photogrammetry to assess the overall abundance and composition of benthic reef communities, including cryptic habitats, are so far lacking.

Cryptic habitats are estimated to account for 75–90% of the volume of a coral reef ecosystem (Choi and Ginsburg 1983; Ginsburg 1983), and up to 8 m^2^ of additional substrate can be found underneath each projected m^2^ of reef surface (Richter et al. 2001; Scheffers et al. 2004). Cryptic substrates are generally densely populated by a distinct benthic community comprised of crustose coralline algae (CCA), encrusting sponges, bryozoans, tunicates, and polychaetes (e.g., Winston and Jackson 1984; Wunsch 1999; Scheffers et al. 2004; van Duyl et al. 2006). To the best of our knowledge, there is only a single assessment of cryptic surface areas at reef scale (Richter and Wunsch 1999; Richter et al. 2001) combined with associated coelobite community sizes (Wunsch 1999) from one geographic region (Northern Red Sea) conducted two decades ago. These seminal studies were limited to accessible cavities, ignoring communities in smaller cryptic spaces, such as the undersides of plating corals (Jackson and Winston 1982). Further, they lack biomass estimates of constituent species needed to study functional interactions between cryptic and exposed reef communities, essentially rendering cryptic spaces the ‘largest, but least known habitat on coral reefs’ (de Goeij and Van Duyl 2007). Cryptic habitats act as major sinks of particulate (Richter and Wunsch 1999) and, predominantly, dissolved organic matter (de Goeij and Van Duyl 2007) and can provide a source of inorganic nutrients that sustain productivity of nearby cryptic and exposed reef organisms (Rasheed et al. 2002; Scheffers et al. 2004; de Goeij et al. 2013), increasing the diversity of reef communities as a whole (Slattery et al. 2013). Comprehensive data on the composition of the entire benthic reef community, including cryptic habitats, are therefore needed to better understand the functional dynamics on coral reefs (Chapin et al. 1997; McCauley et al. 2018).

At present, we do not know to what extent 2D-projections of 3D organisms and the exclusion of cryptic communities in benthic reef surveys influence estimates of the relative abundances and biomass distributions of benthic reef organisms. Therefore, we created an overall census of all major sessile benthic taxa (i.e., scleractinian corals, gorgonians, calcifying algae, non-calcifying phototrophs, massive, and encrusting sponges) in both exposed and cryptic habitats along the leeward fringing reef on the Caribbean island of Curaçao. First, we determined exposed and cryptic surface areas per m^2^ of projected reef by 3D reconstruction. Secondly, we assessed the relative cover of each benthic group using common 2D benthic surveys on horizontal, but also vertical and cryptic reef surfaces. Thirdly, we measured the biovolume of all erect taxa directly in situ. Finally, we produced metric conversion factors for 52 resident species to estimate the biomass of all major sessile benthic groups. This approach enabled us to explore the differences in benthic community composition derived from 2D and 3D benthos surveys. Since different benthic groups perform different ecological functions (e.g., primary and secondary production, calcification, etc.), we aimed to determine a baseline for the benthos on the leeward reef slopes of Curaçao, with potential implications for our overall understanding of the ecosystem functioning of coral reefs.

## Materials and methods

### General survey design and sampling approach

All assessments of benthic communities in both cryptic and exposed reef habitats were conducted from March to June 2018 at 12 reef sites along the leeward shore of Curaçao (Fig. S1, Online resource 1). Sites were chosen to cover a representative range of reef types (healthy vs. degraded, flat vs. complex) along the southwest coast of Curaçao (WAITT-Institute 2017). Based on our sampling design, each site was defined as a 120 m long stretch of reef slope between 9 and 14 m water depth. At each of the 12 sites, two 40-m transects (with 40 m distance between them) were laid out parallel to shore. Measurements and imaging (see below) were conducted in 16 quadrats per site (15 for site Jeremi), spaced apart at 4-m intervals on alternating sides of the two transects. Each quadrat represents all exposed and cryptic reef surfaces within a planar, projected reef area of 1 by 1 m, and was confined by positioning a frame made of PVC pipes. For each quadrat, the water depth and structural relief (i.e., the distance between the lowest and highest points of the reef in contact with the surrounding water) were measured using an Oceanic Veo 3.0 dive computer (0.1 m accuracy). Additional measurements in each quadrat, described in more detail further below, included:

- 3D reconstructions of cryptic and exposed reef surface areas (in m^2^ m^−2^_planar reef_),
- surface area measurements of cryptic surfaces not detected in 3D reconstructions,
- relative cover (in % of total benthos) of the benthic groups occurring on exposed horizontal and vertical, and cryptic reef surfaces,
- volumes of all erect organisms (gorgonians and massive sponges) (in dm^3^ m^−2^_planar reef_),
- canopy heights of non-calcifying phototrophs on exposed horizontal and vertical, and cryptic surfaces.

Additional measurements included tissue thicknesses (in cm) of 30 species and various biomass measures (e.g., ash-free dry weight, organic carbon, in g cm^−2^ or g cm^−3^) of 52 species (Online resource 2). All measures were combined to estimate site-specific (*n* = 16 quadrats per site, except Jeremi where *n* = 15) and island-wide (*n* = 191 quadrats in total) relative abundances of benthic reef organisms in terms of 3D surface area, biovolume, and biomass.

### Surface area estimation of exposed and cryptic habitats

Total reef surface areas within each quadrat were obtained from 3D reconstructions of the reef surface. Our estimates of reef surface area (in m^2^ m^−2^_planar reef_) can be interpreted as measurements of reef rugosity in 3D, although scale differences need to be considered when comparing rugosities determined by different methods. The 3D reconstructions were based on 200–350 overlapping (> 60%) images of all photographable surfaces within each quadrat—excluding small cryptic crevices or deep holes, which were quantified in situ (see below)—using a GoPro HERO6 Black camera (settings: resolution: 4000 × 3000 pixels; aperture: f/2,8; exposure 1/60 s, ISO gain and white balance: automatic) and three GoBe 800-lm video lights to improve uniform illumination of all visible surfaces. We applied a structure-from-motion method for image acquisition and 3D-model generation that was previously established for structural complexity and surface analysis of coral reef 3D reconstructions (Burns and Delparte 2017; Young et al. 2018; Bayley et al. 2019). The 1 × 1 m PVC frames were removed during image acquisition after placing four 15-mL sand-filled Falcon tubes at the corners of each quadrat. Two spirit levels were placed horizontally at a 90° angle on a tripod next to each quadrat to accurately define a horizontal reference plane for the angles of all surface elements in our 3D reconstructions. In addition, the known lengths of the Falcon tubes and spirit levels served as scale bars for the 3D reconstructions.

To generate a 3D representation of all surfaces within each quadrat, (1) images were resized to 2500 × 1875 pixels using the Lanczos resampling algorithm, (2) color adjusted to compensate for site-specific differences in lighting, and (3) an automatic contrast enhancement step was added to facilitate detection and identification of organisms and reef structures. All pre-processing of images was done in XNConvert (https://www.xnview.com/en/xnconvert/). Pre-processed images were exported as JPEG files at maximum compression quality and uploaded in Agisoft Photoscan (Version 1.4; Agisoft, St. Petersburg, Russia; http://www.agisoft.com/downloads/). This program was used to create 3D representations of all visible surfaces in reef quadrats using the following configurations: (1) align photographs: generic image pre-selection, 20,000 key point limit and 8000 tie point limit, (2) dense reconstruction: mild depth filtering, medium density, (3) mesh reconstruction: arbitrary 3D surface, 450,000 faces, and (4) build texture: generic mapping mode, mosaic blend, single texture (4096 × 1). The *marker and rule* tools in Photoscan were used to calibrate all 3D models using the Falcon tubes and the spirit levels for scale. Models were aligned horizontally in reference to the spirit levels and cropped to a 1 by 1 m planar footprint (i.e., 1 quadrat) using the rectangular cropping tool in Photoscan and the four Falcon tubes as reference points. Finally, quadrat models (accuracy: 0.323 ± 0.164 cm, mean ± SD) were saved as Wavefront OBJ files and imported into MeshLab (Cignoni et al. 2008) for surface post-processing and size measurements.

From each 3D-quadrat model, information for each surface element, including surface area, angle, and an estimate of exposure to external observers (i.e., to annotate surface elements to being either cryptic or exposed; see below), was extracted in MeshLab. First, 3D quadrat models were resampled by applying a clustering decimation filter using a cell size of 5 mm to ensure resolution consistency across all reconstructions, after which a two-step Gaussian smoothing filter was applied to remove noise (i.e., spurious 3D points from suspended particles or moving objects). Filter parameters used were: (1) directional bias: 0.5, (2) requested views: 128, (3) lightning direction: [0, 0, 1] (Nadir pointing Sunlight source), and (4) cone amplitude: 60°. Second, the angle (*θ*) of each mesh element toward the water surface was computed as the perpendicular vectors of the element (e.g., *θ* = 0° indicates a plane parallel to the water surface). Third, each element’s exposure to external observers was estimated based on an ambient occlusion algorithm (Landis 2002; Sabbadin et al. 2016, Online resource 1), which is a commonly applied method for 3D models in computer graphics and animations. This algorithm estimates an exposure index for each surface point. The exposure index is defined as the percentage of a full 180° field of view from the surface point that is not occluded by any obstacles, i.e., in a direct line of sight with external observers looking at the reef’s surface (see the drawing in Fig. S2). Thus, an exposure index of 30% implies that the surface point is visible for 30% of all possible sight lines across a 180° field of view, while it is hidden behind obstacles for the remaining 70%. We used the exposure index to categorize modeled reef surfaces as being either cryptic (hidden) or non-cryptic (exposed). For this purpose, we first applied the ambient occlusion algorithm to all surface elements in 3D models of three known exposed (i.e., flat reef tops) and three known cryptic (i.e., cavities) reef surfaces, generating frequency distributions of exposure indices for each of the two surface types (Fig. S3). The frequency distributions of these known exposed and cryptic reef surfaces intersected at an exposure index of 17.5%. Therefore, we choose an exposure index of 17.5% as our threshold value to distinguish between exposed and cryptic surfaces. This implies that cryptic surfaces are ‘out of sight’ for at least 82.5% of all possible sight lines within a 180 degrees field of view.

Exposed surfaces were further distinguished into rather horizontal (0° < *θ* < 45°) and rather vertical (45° ≤ *θ* < 135°) surfaces to account for the fact that communities on these substrates differ (Duran et al. 2018). After analysis, all metrics for exposed horizontal and vertical surfaces were summed to obtain combined estimates of surface areas, volumes, and biomasses for each benthic group on exposed surfaces.

To estimate the total surface area of horizontal and vertical exposed and cryptic reef surfaces, txt files with each element’s surface area, angle, and light index were exported from MeshLab, sorted by surface type and summed in R (RStudio Team 2019; R Core Team 2020). Example illustrations of the individual processing steps to generate 3D reconstructions of each quadrat are provided in Fig. S4.

Small crevices and deep holes inaccessible for our camera did not appear in the photogrammetric 3D reconstructions, so their surfaces and volumes (Online resource 3) were measured by hand in situ and approximated based on formulas of closest known geometrical shapes (i.e., sphere, cylinder, etc.). To account for the lack of fine-scale substrate rugosity associated with geometrical approximations, the ratio of image-based area over hand-measured area was calculated in three accessible caves. The average ratio (1.34 ± 0.10; ± SE) was then used to correct for the underestimation of surfaces from geometric-based cave measurements compared to photogrammetric 3D reconstructions. To quantify cryptic surfaces existing on the underside of sheeting (e.g., *Agaricia* spp.) or at the base of stalking corals (e.g., *Eusmilia fastigiata*, *Madracis mirabilis*), these corals were photographed from several angles (*n* = 13 for *Agaricia *spp. and *n* = 18 for stalking corals). The images were scaled in ImageJ (version 1.X) (Schneider et al. 2012) to estimate surface areas of live coral and cryptic substrate using the *framing tool*. The ratio of cryptic substrate area to live coral area yields the proportional cryptic surface at the undersides of sheeting corals (57 ± 4% of live sheeting coral surface) and bases of stalking corals (231 ± 26% of live stalking coral surface, Online resource 4), which we then applied to our 3D surface area estimates for these corals to calculate the cryptic surface underneath these corals. Total cryptic surface area was estimated by adding these additional cryptic surfaces to our 3D reconstructed cryptic surfaces. Since smaller and highly inaccessible cavities and crevices could not be accessed, we acknowledge that our estimates of cryptic surface areas must be considered underestimations and are therefore conservative.

### Relative cover on exposed and cryptic reef surfaces

Based on their taxonomic identity and morphological growth form, the sessile benthic species were classified into gorgonians, massive sponges, massive corals, branching corals, encrusting corals, foliose corals, sheeting corals, stalking corals, solitary corals, encrusting sponges, benthic cyanobacterial mats, macroalgae, turf algae, crustose coralline algae, *Halimeda* spp.,  *Peyssonnelia* spp*.*, bryozoans, hydrozoans, bivalves, polychaetes, and tunicates, (see Tables S1 and S2 for individual species and group allocations; see Online resource 5 for raw cover data). Sponges were identified to the lowest taxonomic level possible based on photographs and field surveys. The species were further aggregated into seven major benthic groups: gorgonians, massive sponges, scleractinian corals, encrusting sponges, non-calcifying phototrophs, calcifying algae, and others (Table 1).

**Table 1:** Total abundances of benthic reef residents in terms of 2D relative cover, 3D surface area, biovolume, biomass (ash-free dry weight), and organic carbon per m^2^ of planar reef, summed over both exposed and cryptic surfaces

Organism	2D Cover (%)	3D Surface area (dm^2^ m^−2^_planar reef_)	Tissue Volume (dm^3^ m^−2^_planar reef_)	Ash-free Dry weight (g m^−2^_planar reef_)	Organic Carbon (g m^−2^_planar reef_)
**Gorgonians**	2.72 ± 0.34	5.49 ± 2.32	0.94 ± 0.14	106 ± 33.8	47.9 ± 16.0
**Massive sponges**	1.52 ± 0.29	4.36 ± 1.80	3.53 ± 1.24	284 ± 102	126 ± 45.2
HMA sponges	1.18 ± 0.26	2.21 ± 1.55	2.65 ± 1.21	261 ± 121	116 ± 54.1
LMA sponges	0.11 ± 0.04	1.60 ± 0.89	0.30 ± 0.23	10.7 ± 8.29	4.83 ± 3.75
*Agelas clathrodes*	0.52 ± 0.16	0.82 ± 0.96	0.64 ± 0.25	61.8 ± 27.7	28.5 ± 12.6
*Agelas conifera*	0.02 ± 0.01	0.14 ± 0.22	0.02 ± 0.02	1.24 ± 1.24	0.55 ± 0.55
*Agelas sventres*	0	0.11 ± 0.15	0	n.d	n.d
*Aiolochroia crassa*	0.04 ± 0.02	0.04 ± 0.03	0.17 ± 0.11	37.8 ± 25.1	17.4 ± 11.6
*Aplysina archeri*	0.07 ± 0.04	0.07 ± 0.04	0.12 ± 0.09	12.9 ± 10.1	6.01 ± 4.70
*Aplysina cauliformis*	0.02 ± 0.02	0.06 ± 0.11	0.01 ± 0.01	1.77 ± 1.77	0.82 ± 0.82
*Aplysina lacunosa*	0.16 ± 0.09	0.17 ± 0.10	0.19 ± 0.13	22.1 ± 16.0	9.81 ± 7.12
*Biemna* sp.	0	0.01 ± 0.01	< 0.005	0.02 ± 0.02	0.01 ± 0.01
*Callyspongia plicifera*	0.02 ± 0.01	0.08 ± 0.13	< 0.005	0.04 ± 0.02	0.02 ± 0.01
*Callyspongia vaginalis*	0.03 ± 0.02	0.08 ± 0.11	0.01 ± 0.01	0.56 ± 0.33	0.27 ± 0.16
*Desmapsamma anchorata*	0.01 ± 0.01	0.01 ± 0.01	0.01 ± 0.01	0.55 ± 0.54	0.24 ± 0.23
*Ectyoplasia ferox*	0	0	< 0.005	0.06 ± 0.06	0.03 ± 0.03
*Ircinia campana*	0.11 ± 0.05	0.16 ± 0.15	0.12 ± 0.07	12.2 ± 6.98	5.29 ± 3.02
*Ircinia felix*	0.10 ± 0.09	0.39 ± 0.63	0.02 ± 0.01	1.17 ± 0.64	0.45 ± 0.24
*Ircinia strobilina*	0.04 ± 0.02	0.13 ± 0.11	0.10 ± 0.04	5.29 ± 2.11	2.11 ± 0.81
*Neofibularia nolitangere*	0.07 ± 0.07	0.16 ± 0.22	0.07 ± 0.05	3.65 ± 2.50	1.53 ± 1.05
*Niphates erecta*	0.05 ± 0.02	1.42 ± 0.95	0.28 ± 0.23	12.9 ± 10.9	5.70 ± 4.82
*Xestospongia muta*	0.15 ± 0.15	0.16 ± 0.16	1.17 ± 1.17	43.1 ± 43.3	17.2 ± 17.3
Other massive sponges	0.12 ± 0.06	0.35 ± 0.38	0.23 ± 0.10	18.4 ± 8.32	8.14 ± 3.68
**Scleractinian corals**	32.1 ± 1.58	75.3 ± 12.8	1.09 ± 0.25	456 ± 95.8	138 ± 31.0
Massive corals	12.4 ± 0.93	28.3 ± 21.0	0.38 ± 0.30	245 ± 186	78.3 ± 60.1
Branching corals	1.22 ± 0.18	2.91 ± 1.12	0.05 ± 0.03	12.0 ± 6.14	1.85 ± 0.89
Encrusting corals	1.80 ± 0.19	12.2 ± 3.53	0.17 ± 0.07	66.2 ± 29.8	23.5 ± 10.3
Foliose corals	4.28 ± 0.49	8.99 ± 1.93	0.07 ± 0.05	13.5 ± 3.27	1.72 ± 0.43
Sheeting corals	1.29 ± 0.19	3.72 ± 2.26	0.05 ± 0.04	27.6 ± 18.6	10.2 ± 6.79
Stalking corals	11.1 ± 1.34	18.0 ± 11.5	0.33 ± 0.25	149 ± 97.6	30.1 ± 19.9
Solitary corals	0	1.13 ± 0.27	0.02 ± 0.01	6.26 ± 1.63	2.77 ± 0.78
**Encrusting sponges**	0.07 ± 0.03	139 ± 57.3	5.30 ± 2.47	590 ± 299	271 ± 138
*Clathria* sp.	0	0.60 ± 0.42	0.01 ± 0.00	0.39 ± 0.28	0.18 ± 0.13
*Halisarca caerulea*	0	1.81 ± 1.94	0.02 ± 0.02	1.29 ± 1.40	0.57 ± 0.62
*Monanchora arbuscula*	0	0.99 ± 0.54	0.01 ± 0.00	0.68 ± 0.37	0.30 ± 0.16
*Phorbas amaranthus*	0	0.71 ± 0.57	0.03 ± 0.02	1.82 ± 1.51	0.91 ± 0.75
*Plakortis* sp.	0	0.40 ± 0.50	0.04 ± 0.06	6.23 ± 7.72	2.88 ± 3.57
*Scopalina ruetzleri*	0.05 ± 0.02	4.49 ± 2.03	0.21 ± 0.10	18.1 ± 8.86	7.73 ± 3.80
Other encrusting sponges	0.02 ± 0.02	130 ± 131	4.96 ± 5.13	552 ± 571	253 ± 262
**Non-calcifying phototrophs**	51.6 ± 1.57	176 ± 33.9	3.21 ± 0.32	55.6 ± 10.0	22.8 ± 4.20
Benthic cyanobacterial mats	3.20 ± 0.31	7.44 ± 3.25	0.55 ± 0.24	0.94 ± 0.41	0.38 ± 0.17
Macroalgae	29.1 ± 1.55	96.8 ± 21.2	0.11 ± 0.02	45.1 ± 9.90	19.0 ± 4.16
Turf algae	19.4 ± 1.17	71.9 ± 5.29	2.55 ± 0.20	9.60 ± 1.60	3.40 ± 0.61
**Calcifying algae**	2.99 ± 0.37	129 ± 19.0	0.61 ± 0.21	151 ± 32.1	43.6 ± 11.3
Crustose coralline algae	1.68 ± 0.29	70.1 ± 13.0	0.18 ± 0.03	90.7 ± 22.9	15.7 ± 3.42
*Halimeda* sp.	1.03 ± 0.22	2.98 ± 1.90	0.01 ± 0.01	1.67 ± 1.09	0.55 ± 0.36
*Peyssonnellia spp*	0.28 ± 0.06	56.6 ± 14.5	0.52 ± 0.13	90.9 ± 28.4	36.0 ± 11.0
**Other**	8.59 ± 0.71	10.6 ± 0.81	1.34 ± 0.41	49.4 ± 11.8	21.8 ± 6.65
Bryozoans	0	1.85 ± 0.23	n.d	n.d	n.d
Hydrozoans	0	22.6 ± 1.44	0.67 ± 0.40	31.4 ± 11.6	17.2 ± 6.63
*Lithophaga* sp.	0	8.71 ± 0.73	0.37 ± 0.07	12.5 ± 1.33	2.70 ± 0.28
*Polychaetes*	0.44 ± 0.10	15.7 ± 3.74	n.d	n.d	n.d
*Didemnum* sp.	0	9.53 ± 0.79	0.27 ± 0.04	3.68 ± 0.35	1.10 ± 0.25

Values are mean ± SE. See Tables S1, S2 and *Materials and Methods* for sample sizes of underlying measurements

The percentage of surface covered by different benthic groups was quantified for horizontal and vertical exposed and cryptic surfaces separately, in each of the 191 quadrats. A Nikon Coolpix P7000 (Nikon Corp., Japan) underwater camera was used to first obtain one 1 m^2^ top-view image of each quadrat as in traditional reef surveys (horizontal cover; *n* = 16 images per site). An INON S-2000 strobe (INON Inc., Japan) was used to ensure proper lighting and avoid shadows. Subsequently, for each quadrat, three side-view images of haphazardly determined vertical reef surfaces were made with the same camera equipment to estimate vertical cover (*n* = 48 images per site, with an average surface area of 450 ± 280 cm^2^ (mean ± SD) per image). Whenever possible, we chose one surface parallel to the reef slope, and two surfaces perpendicular to the reef slope: one up- and one down-current to the long-term mean current from SE to NW. Lastly, three cryptic surfaces per quadrat were photographed using an Olympus Tough TG-5 underwater compact camera (Olympus Corp., Japan) to estimate cryptic cover (*n* = 48 images per site, with an average surface area of 118 ± 82 cm^2^ per image). Specifically, for these cryptic surfaces we chose, where possible due to space limitations, one ‘roof’ surface, one ‘side’ surface, and one ‘back’ surface per quadrat. Benthic community composition in each image was analyzed using point-count analyses (80 stratified random points per picture) in Coral Point Count with Excel extension (CPCe) (Kohler and Gill 2006).

Some substrates were covered by communities through which the community on the underlying substrate could still be seen (e.g., turf algae with an understory of CCA). In those cases, only the overlying turf algae was counted in 2D cover estimates, while for all 3D estimates (see below), the percentage bottom cover of both overlying and underlying communities was estimated separately so that total cover estimates exceeded 100% in some cases (see Online resource 5).

### 3D surface area of benthic groups

The 3D surface area of each benthic group per site (SA_*i*_ for benthic group *i*) was first calculated for horizontal and vertical exposed and cryptic surfaces separately. Average substrate surface areas (A, in m^2^ m^−2^_planar reef_) obtained from the 3D reconstruction of the reef were multiplied by the average relative 2D cover (C_*i*_) of a benthic group across all 16 quadrats (or 15 for site Jeremi) per site. For example, the 3D surface area of benthic group *i* on horizontal surfaces (denoted by the subscript ho) was calculated as:

1 $${\text{SA}}_{{{\text{ho}},i}} = {\text{A}}_{{{\text{ho}}}} \cdot {\text{C}}_{{{\text{ho}},i}}$$

Standard errors of these estimates were calculated by propagating standard errors of A and C (shown again for horizontal 3D surface area of benthic group *i*):

2 $$\delta {\text{SA}}_{{{\text{ho,}}i}} = {\text{SA}}_{{{\text{ho}},i}} \cdot \sqrt {\left( {\frac{{\delta {\text{A}}_{{{\text{ho}},i}} }}{{{\text{A}}_{{{\text{ho}},i}} }}} \right)^{2} + \left( {\frac{{\delta {\text{C}}_{{{\text{ho}},i}} }}{{{\text{C}}_{{{\text{ho}},i}} }}} \right)^{2} }$$

where *δ* refers to standard error (SE). Estimates for horizontal and vertical exposed and cryptic surfaces were summed up to obtain total 3D surface area per site for benthic group *i* (TSA, in m^2^ m^−2^_planar reef_), Online resource 6). The standard error of the total surface area was obtained by propagating the standard errors from Eq. [2] according to:

3 $$\delta {\text{TSA}}_{i} = \sqrt {\left( {\delta {\text{SA}}_{{{\text{ho}},i}} } \right)^{2} + \left( {\delta {\text{SA}}_{{{\text{ve}},i}} } \right)^{2} + \left( {\delta {\text{SA}}_{{{\text{cr}},i}} } \right)^{2} }$$

where the subscripts ve and cr denote vertical and cryptic surfaces. In a similar calculation, the total exposed 3D surface area was calculated using only the values for horizontal and vertical reef surfaces. Island-wide 3D surface areas for each benthic group (e.g., group *i*) were calculated using the average surface areas and 2D surface cover across all 12 sites (*n* = 191 quadrats).

### Biovolumes of benthic groups

Biovolumes of erect organisms (i.e., massive sponges and gorgonians) were estimated by dividing each individual into one or more geometrical forms (i.e., rods, cone, sphere, bowls, barrels, see Table S3) that were measured in situ using a measuring tape or ruler, taking into account accessible empty space (e.g., larger oscula of some sponges). This was done because individuals assigned to these groups often moved (in the current) and as a consequence could not be reconstructed in 3D through structure-from-motion techniques. To ensure that all erect organisms were measured in a similar manner, we also hand-measured individuals that did not move in the current. The cuboid geometrical form was used to estimate the biovolume of all non-erect organisms by multiplying their 3D surface area to their average thickness. The thickness of organic tissues for each non-erect species was derived from side-view photographs of broken colonies in ImageJ. Since we considered all calcified framework as substrate (including the 3D structures produced by scleractinian corals), the coral tissue itself represents a thin layer overlying such structures (Edmunds and Gates 2002) and was primarily measured as a surface rather than a volume. The biovolume of non-erect organisms can be estimated by multiplying 3D surface cover to an average tissue height of the respective organism (hence cuboid form). In contrast, erect organisms (e.g., massive sponges, gorgonians) form an individual volume (3D shape) extending from the substrate. An exception from this are non-calcifying phototrophs (i.e., benthic cyanobacterial mats, macroalgae, and turf algae), where canopy heights can vary dramatically and were therefore measured in situ in all quadrats for horizontal and vertical exposed and cryptic surfaces separately (*n* = 3 for each taxon per surface type per quadrat). To calculate biovolumes of turf algae and benthic cyanobacterial mats, average canopy height was multiplied by their average 3D surface area. The estimated volumes of tissue on each surface type were then summed and their errors propagated analogous to Eq. [3] to calculate overall biovolume in dm^3 ^m^−2^_planar reef_.

The dominant macroalgae (*Dictyota* spp. and *Lobophora *spp.) were difficult to discern on CPCe images and therefore assessed as a single benthic group. Further, these algae grow in loose bundles of leaves that can be more or less dense. This means the empirical relationships between canopy height and tissue volume for a given surface area may not be linear, rendering the cuboid calculation potentially erroneous. We therefore first calculated the empirical regression between canopy height and tissue volume for a total of 9 tissue samples of each species covering 25–160 cm^2^ of substrate with varying canopy height (Fig. S5a, b, Table S4). For each sample, thallus surface area was estimated by laying out all thalli on a white sheet and taking scaled images to be analyzed for total surface area in ImageJ. The tissue volume of these samples was calculated by multiplying thallus surface area with average thallus thicknesses of 70 µm for *Dictyota* and 40 µm for *Lobophora*, respectively (Tronholm et al. 2010; Vieira 2015; Camacho et al. 2019), and then regressed against the sampling area. The average in situ canopy height of macroalgae was inserted (*x*-value) into these empirical regressions to calculate conversion factors (i.e., *y*-values in Fig. S5a, b) from 3D surface area to volume for that quadrat and surface type (see Online resource 1 for specific equations). Empirical regressions differed between *Dictyota *spp*.* and *Lobophora *spp*.*, and the average conversion was used to calculate the biovolume of macroalgae. Total and exposed biovolumes for all benthic groups were obtained by summing estimates of volumes on horizontal and vertical surfaces with and without cryptic surfaces, respectively, as described above for 3D surface area.

### Biomass of benthic groups

Tissue samples of various sizes (0.7–20 cm^3^ for erect organisms, 1.7–614 cm^2^ for non-erect organisms, see Online resource 2 for exact sizes) were collected for representative species of all benthic groups (*n* = 3–8 samples per species) (see Tables S1, S2 in Online resource 1 for detailed species list and sample sizes) across the surveyed area using different approaches specific to each group (see below). Tissue samples were collected to estimate tissue thickness, ash-free dry weight (AFDW), organic carbon (C_org_), and organic nitrogen (N_org_). Samples rich in calcium carbonate (e.g., scleractinian corals, crustose coralline algae) were collected by chiseling out fragments containing the entire tissue layer (and some underlying calcium carbonate) and subsequently stored in individual 50-mL Falcon tubes. Samples of organisms with embedded skeletal structures (e.g., gorgonians, sponges) were collected by carefully scraping or cutting off a tissue area or volume of known size, using a sharp blade. Sampling of epibionts was avoided and samples were stored in 15-mL falcon tubes. Non-calcifying phototrophs and soft coelobites (e.g., *Didemnum* or hydrozoans) were carefully collected using tweezers and stored in zip lock bags. All samples were photographed in situ and transported to the lab for processing within 2 h, where they were photographed again next to a scale. Samples were quickly rinsed in deionized and distilled water (18.2 MΩ−cm type I, Elga Purelab Classic UV) to remove excess salts, and subsequently freeze-dried (Scanvac Coolsafe 55-4, Labogene). Sample dry weight was determined on a precision scale (± 0.01 mg), after which samples were homogenized (Planetary Ball Mill Pulverisette 5, Laval Lab) for 8 min, at a relative centrifugal force of 22×*g*, and divided equally into three aliquots. One aliquot was weighed, combusted at 450 °C for 4 h, and weighed again to determine AFDW. The remaining two aliquots were both acidified to remove inorganic C using 4 mol L^−1^ hydrochloric acid until effervescence ceased (Nieuwenhuize et al. 1994) and analyzed for C_org_ and N_org_ (duplicate measurements) on a carbon, hydrogen, nitrogen, sulfur elemental analyzer (CHNS-EA; Elementar Vario El Cube). H and S contents were not extracted from the output file.

Images of samples were uploaded into ImageJ to estimate surface area and volume using delineation of approximated geometrical shapes (see above). Conversion factors to CT-scanned surface area estimates (Naumann et al. 2009) were applied to samples of corals with grooved or folded surfaces (Online resource 2) to avoid scale differences with the 3D reconstructions used to extrapolate biomass to reef-scale. These corals included *Acropora cervicornis* (factor = 0.95)*, Agaricia *spp., *Mycetophyllia *spp., and *Pseudodiploria strigosa*. The latter three species were converted using the ratio determined for the morphologically most similar species *Montipora *spp. (factor = 1.37, Naumann et al. 2009). Smaller irregularities in tissue samples of other non-erect reef organisms did not lead to scale differences with 3D models, except in non-calcifying phototrophs.

To calculate the biomass of non-calcifying phototrophs, we used the same approach as described above for the biovolume of macroalgae, converting 3D surface area to biomass using the empirical regression between canopy height and AFDW for a given area (Fig. S5c–f for macroalgae and Fig. S6 for turf algae and benthic cyanobacterial mats). For all erect organisms, whose volumes were measured directly in situ (Online resource 7), biomass was estimated by volume-to-biomass conversion factors (Table S1) multiplied by their in situ-measured biovolumes. For encrusting sponges, corals, crustose coralline algae, and other coelobites (e.g., *Didemnum *spp*.*, hydrozoans), biomass was estimated by surface-to-biomass conversion factors (Table S2) multiplied by their 3D surface areas (Online resource 8). The same calculations (see Online resource 1) were used to estimate standing stocks of organic carbon (Online resource 9) and nitrogen (Online resource 10), but using weight estimates for C_org_ and N_org_, respectively (Figs S7, S8), rather than AFDW.

Final abundance metrics (3D surface area, biovolume, biomass) for the seven major benthic groups (Table 1) were generated by summing estimates for each benthic group (see Tables S1, S2 in Online Resource 1 for the applied classification), and uncertainties were once again computed analogous to Eq. [3]. All raw data, equations, and summary statistics are provided in Online resources 1–11.

### Statistical analysis

Since relief is commonly used as proxy for structural complexity on reefs, we tested whether relief predicts our measured surface areas (i.e., the summed area of all surfaces present within the reef volume underneath a 1 × 1 m projected reef surface). All data were log transformed prior to analysis to obtain normal distributions (Fig. S9). Simple linear regression was used with relief as predictor variable. Dependent variables were either cryptic, exposed, or total surface area per quadrat. Results were then back-transformed for plotting. A roughly uniform spread of residuals verified homoscedasticity of the underlying data.

## Results

We characterized the composition of reef communities between 9 and 14 m water depths along the leeward shore of Curaçao in terms of relative 2D and 3D benthic cover, biovolume, and biomass. We found that surveys taking into account the three-dimensional nature of coral reefs and reef organisms, and using biomass rather than cover as an abundance metric, greatly altered the relative contribution of dominant benthic groups to overall community composition.

### Reef 3D surface areas

**Fig. 1:**
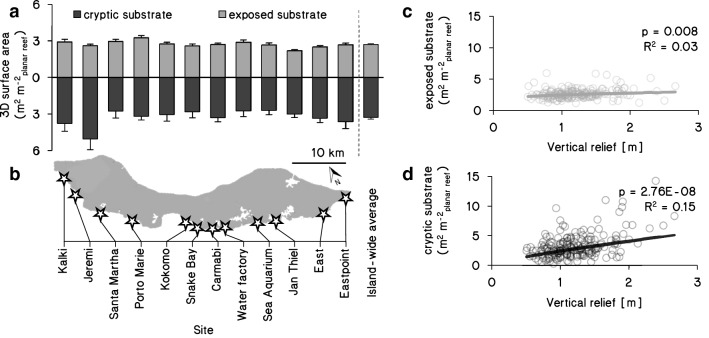
Available 3D-projected surface area in exposed and cryptic habitats along the leeward fringing reefs of Curaçao. Exposed (gray bars, positive y-axis) and cryptic (black bars, negative *y*-axis) surface areas at 9–14 m depth (**a**) are shown for 12 sites on Curaçao (**b**). Bars represent mean values (± SE) in m^2^ m^−2^_planar reef_ for each site (based on *n* = 16 quadrats, except Jeremi where *n* = 15), and all sites combined (*n* = 191). Also shown are correlations between exposed (**c**) or cryptic (**d**) substrate (*y*-axis) and vertical relief (i.e., distance between highest and lowest points in contact with seawater, *x*-axis) for all quadrats (n = 191)

The average amount of reef surface area (in m^2^ m^−2^_planar reef_) per site ranged from 5.2 ± 0.4 (mean ± SE,* n* = 16 quadrats, Jan Thiel) to 7.6 ± 1 (*n* = 15, Jeremi; Fig. 1). The island-wide average was 6.0 ± 0.2 m^2^ m^−2^_planar reef_ (*n* = 191 quadrats in 12 sites). Approximately half of the total reef surface area consisted of cryptic surfaces (island-wide average: 3.3 ± 0.2 m^2^ m^−2^_planar reef_). The maximum reef surface observed within a quadrat was located at Jeremi and reached 17.4 m^2^ m^−2^_planar reef_, of which 82% was cryptic surface. Relief as a proxy for reef flatness correlated significantly with total surface area, but explained only 16% of the variation (Pearson correlation: *R*^2^ = 0.16, *n* = 191, *p* < 0.001; Fig. S9a, b). This correlation was stronger for cryptic substrates (*R*^2^ = 0.15, *n* = 191, *p* < 0.001, Fig. 1d) than for exposed substrates (*R*^2^ = 0.03, *n* = 191, *p* < 0.01, Fig. 1c).

### Reef benthic community composition

**Fig. 2:**
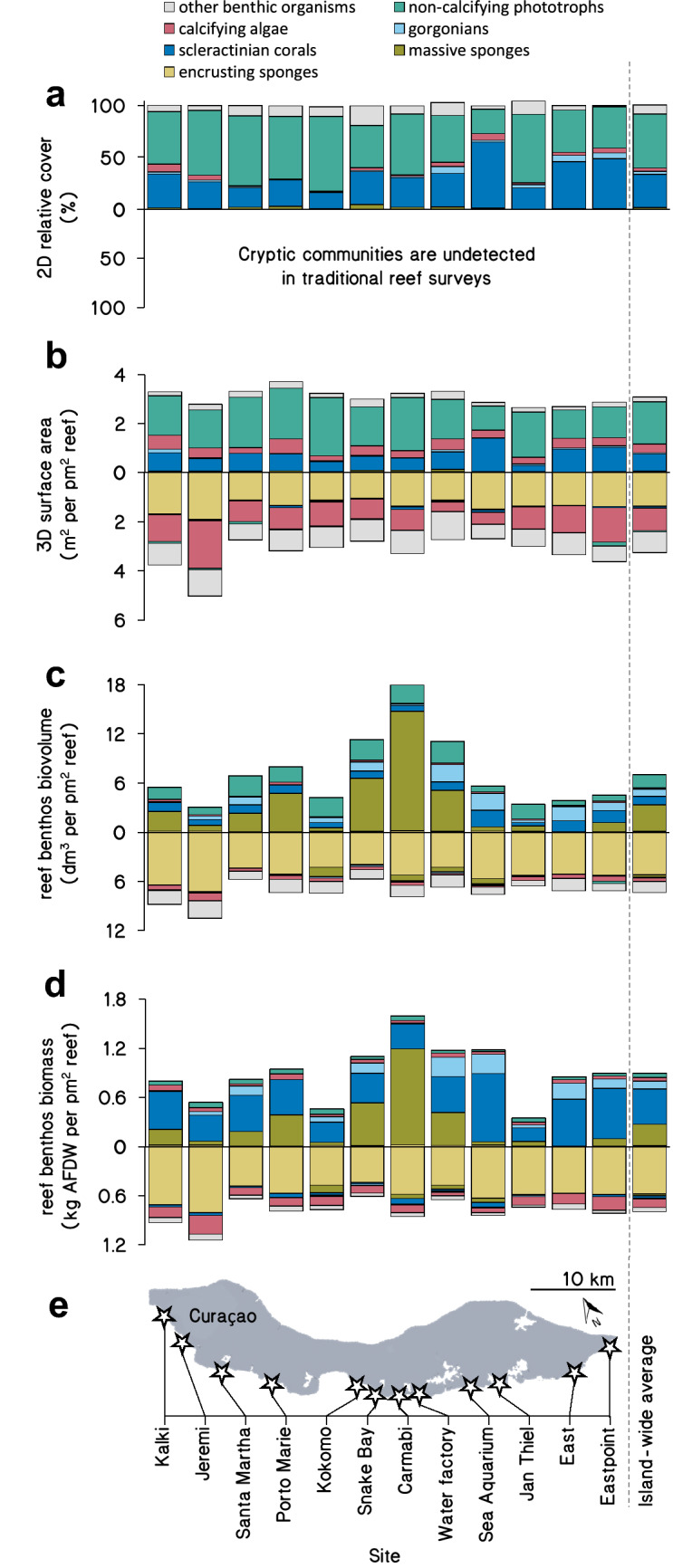
Size and composition of the reef benthos using different 2D and 3D abundance metrics. Mean community sizes (± SE) in exposed (positive *y*-axes) and cryptic (negative *y*-axes) reef habitats at 9–14 m depth at 12 sites along the leeward shore of Curaçao are shown as 2D relative cover in % of total benthos (**a**), 3D covered surface area in m^2^ m^−2^_planar reef_ (**b**), reef benthos biovolume in dm^3^ m^−2^_planar reef_ (**c**), and reef benthos biomass in kg ash-free dry weight (AFDW) m^−2^_planar reef_ (**d**) along the leeward shore of Curaçao (**e**). *n* = 16 quadrats per site, except Jeremi where *n* = 15. SE’s are provided in Table 1. In panel (**a**), the category ‘other benthic organisms' also includes exposed sediment and rubble

The estimated contributions of different benthic groups to the total reef benthos greatly depended on the abundance metric used (2D/3D cover, biovolume, or biomass) (Fig. 2, Table 1).

#### 2D projected cover

**Fig. 3:**
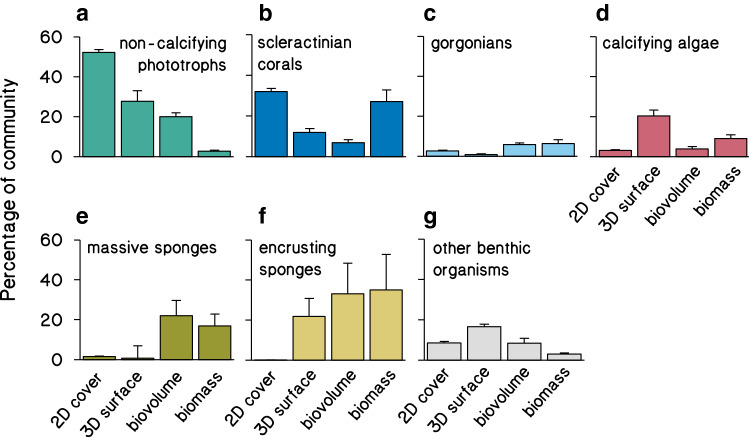
Relative contributions of benthic groups using different 2D and 3D abundance metrics. Shown are relative proportions (mean ± SE) of each benthic group to the total benthic reef community size in terms of 2D relative cover, 3D surface area, biovolume, and biomass (*n* = 191 quadrats, see Tables S1 and S2 for sample sizes of metric conversions)

According to a traditional assessment, using the percent relative cover estimated by 2D projection of exposed reef surfaces, the most abundant benthic groups were non-calcifying phototrophs (52 ± 2%) and scleractinian corals (32 ± 2%) (Figs. 2a, 3a, b; Table 1). Lowest cover was found for encrusting sponges (0.07 ± 0.03%; Fig. 3f) and massive sponges (1.5 ± 0.3%; Fig. 3e). Note that this traditional 2D relative cover approach does not include benthic communities in cryptic reef habitats and only assesses the exposed reef surfaces visible from photographs taken above the reef (Fig. 2a).

#### 3D total cover

When considering the relative contribution to 3D (exposed and cryptic) reef surface areas, non-calcifying phototrophs were still the largest benthic group (25 ± 5% of total 3D surface area) and dominated the exposed reef surface together with scleractinian corals (11 ± 2%) (Figs. 2b, 3a, b; Table 1). The second and third largest benthic groups were encrusting sponges (20 ± 8%) and calcifying algae (19 ± 3%) that dominated the reef’s cryptic surface areas (Figs. 2b, 3d, f; Table 1). The contribution of organisms with soft, erect morphologies (i.e., massive sponges and gorgonians) to 3D surface area was minor in all reef habitats across the island (Figs. 2b, 3c, e).

**Table 2:** Total benthic community size in terms of 3D surface area, biovolume, biomass (ash-free dry weight), and organic carbon

Habitat		3D surface area (m^2^ m^−2^_planar reef_)	Tissue volume (dm^3^ m^−2^_planar reef_)	Ash free dry weight (kg m^−2^_planar reef_)	Organic carbon (kg m^−2^_planar reef_)
Total reef		6.36 ± 0.81	16.02 ± 2.83	1.69 ± 0.33	0.67 ± 0.15
Exposed reef	abs	3.10 ± 0.19	8.60 ± 1.28	0.90 ± 0.12	0.33 ± 0.05
	rel	0.49 ± 0.03	0.54 ± 0.08	0.53 ± 0.07	0.49 ± 0.08
Cryptic reef	abs	3.26 ± 0.13	7.42 ± 1.24	0.79 ± 0.17	0.34 ± 0.08
	rel	0.51 ± 0.02	0.46 ± 0.08	0.47 ± 0.10	0.51 ± 0.12

Values are mean ± SE. Shown are absolute values (abs) and relative proportions (rel) of exposed (i.e., exposed to sunlight) and cryptic reef communities. See Tables S1, S2 and *Materials and Methods* for sample sizes of underlying measurements

#### Biovolume

When community abundances are expressed as biovolumes, massive sponges (22 ± 8% of total biovolume) and gorgonians (6 ± 1%) became profoundly more dominant on the exposed reef surface (Fig. 2c, Table 1). The contributions of non-calcifying phototrophs (20 ± 2%) and scleractinian corals (8 ± 2%) decreased in comparison to 2D cover and 3D surface area measurements (Figs. 2a–c, 3a, b; Table 1). The biovolume of massive sponges displayed considerable variation among sites, with the highest biovolume at Carmabi in the center of the island and lowest biovolume on the Eastern side. The biovolume of the cryptic community was dominated almost exclusively by encrusting sponges (33 ± 15%), which showed a more consistent abundance across the island (Fig. 2c).

#### Biomass

When expressed as biomass, the reef community composition was generally similar to the reef’s community composition expressed as biovolume (compare Fig. 2c, d). The most clear difference between biomass and biovolume estimations is the increase in contribution of scleractinian corals (27 ± 6% of total biomass) and the diminished contribution of non-calcifying phototrophs (< 5%) to total benthic reef biomass (Figs. 2c, d 3a, b; Table 1). Notably, more than half of the reef biomass was comprised of encrusting (35 ± 18%) and massive (17 ± 6%) sponges (Fig. 3e, f).

The composition of reef communities in terms of organic carbon closely resembled our findings for biomass (Table 1) and is therefore not explicitly plotted or discussed. Volume-to-biomass and surface-to-biomass conversions are provided in Fig. S7, Table S1 and Fig. S8, Table S2, respectively. Additional data underlying our results (e.g., site coordinates, canopy heights of non-calcifying phototrophs, relative cover on different surface types, species-specific biomass conversions, standing stocks and tissue contents of organic nitrogen) are provided in Online resources 2–11.

## Discussion

### The divided community structure of coral reef frameworks

Coral reefs form some of the most diverse ecosystems of our planet, and their biodiversity and ecosystem functioning is severely threatened by coastal eutrophication and climate change (Hughes et al. 2003; Hoegh-Guldberg et al. 2007; Carpenter et al. 2008). Yet, progress in understanding their ecosystem functioning has been strongly limited by a lack of quantitative data on the relative abundances of different functional groups in these complex ecosystems. This study represents a comprehensive assessment of the community composition and size on both the exposed and cryptic surfaces in a coral reef ecosystem. Our results show that approximately half of the total reef surface area, biovolume, and biomass of the fringing reefs of Curaçao resides in ‘hidden’, cryptic spaces (Figs. 1, 2, Table 2). Important to note is that the composition of the benthic groups in this cryptic habitat is markedly different from the exposed part of the ecosystem. Although very few studies have been conducted on cryptic reef communities, Caribbean (Jackson and Winston 1982; Scheffers et al. 2010; van Duyl et al. 2006), Red Sea (Wunsch 1999; Richter et al. 2001) and Indonesian reefs (de Goeij and Van Duyl 2007) show a consistent dominance of encrusting sponges, calcifying algae, and other suspension feeders (e.g., bivalves, hydrozoans, polychaetes, tunicates). These cryptic communities act as major regenerators of organic (Richter et al. 2001; de Goeij and Van Duyl 2007; de Goeij et al. 2013) and inorganic nutrients (Tribble et al. 1990; Gast et al. 1998; Rasheed et al. 2002) within the oligotrophic reef ecosystem. Consequently, the lack of integration of the cryptic habitat in reef-scale assessments seriously hampers our understanding of coral community composition, species interactions, biogeochemical cycling, and thus our understanding of overall coral reef ecosystem functioning in changing oceans. Proper baselines for cryptic reef communities are also needed because coral reefs worldwide are reportedly flattening (Alvarez-Filip et al. 2009; Newman et al. 2015; Bellwood et al. 2018; Magel et al. 2019; Tebbett et al. 2019). Given that a reduction in reef relief is associated with a decrease in cryptic reef surfaces (Fig. 1d), our findings suggest that ongoing flattening of reefs could primarily diminish the largely undescribed cryptic habitats. Further, the lack of correlation between relief and exposed surface area (Fig. 1c) implies that traditional monitoring of reef rugosity could fail to detect this decrease in structural complexity.

### Key insights and implications

**Fig. 4:**
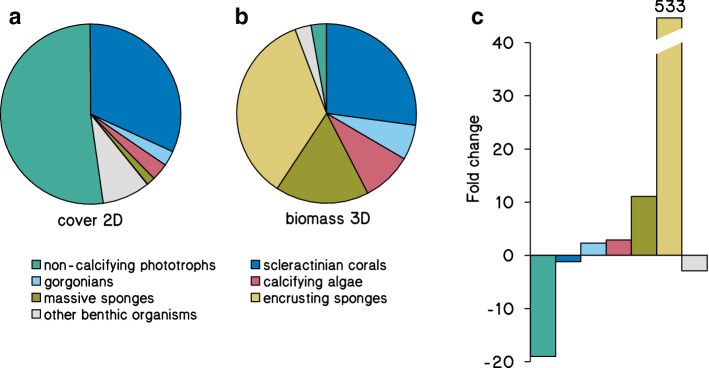
Relative composition of the benthic reef community in terms of cover and biomass. Shown are relative proportions of different benthic groups to benthic reef community cover in 2D (**a**), and whole reef community biomass in 3D (**b**), as well as the fold change from cover to biomass (**c**) (*n* = 191 quadrats)

Including the cryptic habitat in reef assessments indicates that most reef biomass in our study area is represented by sponges, with other major contributions to community biomass by scleractinian corals, gorgonians, and calcifying algae (Figs. 3, 4b; Table 1). Sponges, however, cover only 2% of all exposed substrates along the reef slopes of Curaçao at the surveyed depths (Online resource 6) and are therefore largely overlooked in traditional 2D reef surveys (Figs. 2a, 4). Community biomass on the exposed part of the reef is mainly dominated by massive sponges (Fig. 2d, positive *y*-axis), whereas encrusting sponges dominate the cryptic habitat (Fig. 2d, negative *y*-axis) and can represent a staggering 35% of the total biomass in benthic reef communities (Fig. 3f). This number coheres with recent studies describing how encrusting sponges drive large and important nutrient fluxes between cryptic and exposed reef habitats (de Goeij et al. 2013; Rix et al. 2016; Lesser and Slattery 2020). Specifically, they turn dissolved organic carbon, the largest source of organic matter, into consumable detritus entering the food web, a pathway called the sponge loop (de Goeij et al. 2013). Cycling rates through this sponge loop were estimated to be comparable to overall coral reef primary production. These fluxes were based solely on carbon cycling through cryptic, encrusting sponges under the assumption that 1 m^2^ of reef concealed 2.8 m^2^ of cryptic surface (de Goeij and Van Duyl 2007), which is very close to the island-wide 3.3 m^2^ m^−2^_planar reef_ determined in this study. The extent of dissolved organic carbon cycling through massive (i.e., non-encrusting) sponges and the overall ecological role of sponges under changing ocean conditions are still under debate (e.g., de Goeij et al. 2017; McMurray et al. 2018; Pawlik and McMurray 2020), which illustrates the current lack of knowledge on these hitherto largely neglected key ecosystem drivers.

The biomass of non-calcifying phototrophs was an order of magnitude lower than scleractinian corals despite dominant macroalgal (2D) surface cover (Fig. 4). This was caused foremost by low tissue weights of macroalgae, turf algae, and benthic cyanobacterial mats compared to other benthic taxa (Odum and Odum 1955; Hatcher 1988; Miller et al. 2003). For instance, the AFDW of the heaviest alga *Lobophora *spp*.* covering one square meter of reef substrate was only 70 g m^−2^ and contained 30 g m^−2^ of organic carbon, which is an order of magnitude lower than most other benthic organisms (Table S2). Miller et al. (2003) compared relative cover and biomass of macroalgae in the Florida Keys in 1998 and 1999, reporting 20 and 65% cover, and biomasses of 16 and 60 g m^−2^_planar reef_, respectively. Our estimates for macroalgae (29% cover; 45 g m^−2^_planar reef_), turf algae (19% cover; 10 g m^−2^_planar reef_), and all non-calcifying phototrophs (52% cover; 56 g m^−2^_planar reef_) fall closely within these ranges (Table 1). Importantly, the canopy height of these phototrophs can vary dramatically depending on nutrient loads and grazing pressures (Lapointe et al. 1997; Littler et al. 2006), which affects their biomass but not surface cover. Although we incorporated variation in canopy height in our analysis, it had limited influence on the low contribution of non-calcifying phototrophs to overall reef community biomass, particularly for *Lobophora spp.* (Fig. S5d, f, but see Steneck and Dethier 1994). The relatively low biomass but high cover of non-calcifying phototrophs is accompanied by major contributions of macroalgae to reef-wide primary production (Wanders 1976; Hatcher 1988) and nutrient cycling (Haas et al. 2010; Mueller et al. 2014). The co-occurrence of a high productivity but low biomass is likely due to the high turnover rates of macroalgae, turf algae and benthic cyanobacterial mats, as they are often heavily grazed (Ferrari et al. 2012) and can release substantial amounts of dissolved organic matter (Mueller et al. 2014; Brocke et al. 2015). Hence, as their turnover rates cannot be estimated from snapshots of community composition, the low biomass contributions of non-calcifying phototrophs do not imply that they play only a minor role in ecosystem productivity.

### Surveying reefs in 3D

The abundance metrics portrayed here have different strengths and weaknesses. Organic biomass and element analysis (e.g., C, N, P) estimates—though limited in scaling opportunity—provide the most comprehensive insights into the standing stocks and biogeochemical cycling of energy and (in)organic nutrients in both aquatic and terrestrial ecosystems (Diaz and Rützler 2001; Dubinsky and Stambler 2010; Atkinson 2011; Le Toan et al. 2011). The assessment of coral reef community biovolume requires less effort, but introduces additional challenges, such as measuring the average protrusion of coral tissue into the underlying skeleton, empty spaces in tissues of massive sponges, or the free space between filaments of turf algae and benthic cyanobacterial mats. All are subject to assumptions in this study and would need improved measures to gain accuracy and confidence. At the other end of the spectrum, traditional 2D surveys are fast, cost-effective, and scalable (Gardner et al. 2003; De’ath et al. 2012), but incomplete to varying degrees depending on the reef’s structural complexity. While we acknowledge that many reef assessments span much larger survey areas compared to this study, we support previous recommendations to assess reefs in 3D (González-Rivero et al. 2014; Burns et al. 2015; Ferrari et al. 2016). This step is substantially facilitated by the recent surge in studies validating structure-from-motion photogrammetry as an appropriate tool to generate digital twins of reefs (Figueira et al. 2015; Leon et al. 2015; Guo et al. 2016; Burns and Delparte 2017). We show that 3D surface areas can be estimated from these models, automatically assigned into exposed (i.e., visible) and cryptic (i.e., occluded) reef surfaces, and combined with traditional coral point counts on these surfaces to estimate 3D surface cover. However, for trophic modeling, 3D surface cover alone is still not sufficiently representative for the contribution of individual taxa to reef communities (Fig. 2b, d). Community data can be converted to biochemical proportions using conversion factors from the extending literature from several worldwide coral reef locations (Brey et al. 2010; Stratmann et al. 2020). Adopting such a strategy would require relatively little additional effort, while dramatically increasing the ecological resolution of ongoing accounts of coral reef communities.

### Limitations and conclusion

There are some important limitations regarding our biomass estimates. First, they must be considered conservative estimations, since despite our best efforts to quantify all existing benthic organisms, some biota residing within inaccessible cavities and endolithic taxa, such as bioeroding sponges or other burrowing organisms, were not included due to logistical constraints and to avoid destructive practices associated with such measurements (Fang et al. 2013). Complementary approaches, such as the use of artificial substrates (Leray and Knowlton 2015; Vicente et al. 2021), could greatly improve the identification and quantification of these cryptic communities. Second, cryptic sediments were not quantified in this study. Although exposed sediment cover was low (5.7%) in our survey areas (Online resource 6), cryptic sediments can account for up to 40% of the substrate in coral reef cavities (de Goeij and Van Duyl 2007), while data on the standing stocks of local sediment micro- and macrofaunal communities are lacking. Third, we acknowledge that our sampling approach (e.g., the selection of surfaces for vertical and cryptic reef assessments) may have caused biases in our community estimations and further improvements in randomization of the spatial sampling methods are needed to monitor changes in 3D reef communities over time (e.g., Smith et al. 2017). Our data therefore serve as a first example of biomass distributions in Caribbean reefs or even tropical coral reefs in general. Here described patterns of sponge-dominance are in line with previous reports from both exposed (Pawlik 2011; Bell et al. 2013) and cryptic reef habitats (Meesters et al. 1991; Scheffers et al. 2010), including Red Sea reefs (Richter and Wunsch 1999). Yet, a variety of geographical areas need to be surveyed using similar approaches to validate our finding that sponges dominate standing stocks of benthic biomass on Caribbean coral reefs (Rützler 1978). Lastly, motile fauna and pelagic biota represent important components of coral reefs that were not assessed in this study. While community data on motile invertebrate abundance do not exist for the studied area, local fish biomass has been estimated to account for 0.130 ± 0.012 kg m^−2^_planar reef_ (Sandin et al. 2008a). Bacterio- and phytoplankton standing stocks on local reefs amount to < 0.001 kg m^−2^_planar reef_ (Lesser et al. 2020), assuming an average water depth of 10 m. While these pelagic communities are one to four orders of magnitude smaller than the benthic communities determined in this study (1.7 ± 0.3 kg m^−2^_planar reef_), they should also be incorporated into standing stock assessments, along with motile invertebrates, to improve our understanding of how benthic-pelagic interactions shape marine ecosystems and their response to environmental change.

We show that different abundance metrics (2D cover, 3D surface area, biovolume, biomass) lead to markedly different perspectives on benthic reef community composition. These different abundance metrics serve different ecological questions. For example, conventional 2D approaches may provide the best balance between accuracy, scale, and required resources if the goal is to indicate general reef health based on the relative proportions of, for example, scleractinian corals and macroalgae. But the same conventional approach structurally overestimates the biomass contribution of conspicuous primary producers (i.e., macroalgae, turf algae), while underestimating the contributions of reef taxa with erect morphologies (i.e., gorgonians, massive sponges) that have important functions in nutrient cycling, biodiversity, and reef productivity (Ferrier-Pagès and Gattuso 1998; Maldonado et al. 2012; de Goeij et al. 2017). To move beyond limited descriptions of the current state of reefs and incorporate (biogeochemical) processes driving reef states, newly emerging 3D approaches using photogrammetry (González-Rivero et al. 2014; Burns et al. 2015; Ferrari et al. 2016) mostly overcome this limitation, albeit they do not capture the cryptic habitat specifically, potentially rendering large parts of the reef system undetected, such as crevices and holes not obvious to an observer swimming over a reef. Biomass and organic carbon stocks determined in this study will strengthen estimates of ecosystem productivity and biogeochemical cycling in coral reefs, and our metric conversions can be used to augment reef surveys at other Caribbean locations, to ultimately improve predictions of how complex benthic ecosystems develop in the Anthropocene.

## Supplementary﻿ Information

Below is the link to the electronic supplementary material. Online resource 1 (DOCX 3086 kb) Online resource 2 (XLSX 89 kb) Online resource 3 (XLSX 50 kb) Online resource 4 (XLSX 9 kb) Online resource 5 (XLSX 509 kb) Online resource 6 (XLSX 96 kb) Online resource 7 (XLSX 107 kb) Online resource 8 (XLSX 74 kb) Online resource 9 (XLSX 74 kb) Online resource 10 (XLSX 74 kb) Online resource 11 (XLSX 30 kb)
