# Supplementary material for: Implications of 2D versus 3D surveys to measure the abundance and composition of benthic coral reef communities
Source: Coral Reefs. 2021 Jun 16;40(4):1137–53. doi: 10.1007/s00338-021-02118-6 (PMC8550779; doi:10.1007/s00338-021-02118-6)
Supplement: Supplementary file 1 — Online resource 1 (DOCX 3086 kb) [file 338_2021_2118_MOESM1_ESM.docx]

Supplementary Information for:

‘Implications of 2D versus 3D surveys to measure the abundance and composition of benthic coral reef communities’

Niklas A. Kornder, Jose Cappelletto, Benjamin Mueller, Margaretha J. L. Zalm, Stephanie J. Martinez, Mark J. A. Vermeij, Jef Huisman, Jasper M. de Goeij

Niklas A. Kornder

Email: n.kornder@web.de

**This PDF file includes:**

Figures S1 – S9

Tables S1 – S4

Supplementary texts 1 & 2

Captions of additional supplementary materials:

Online resources 2 – 11

3D models 1 – 191


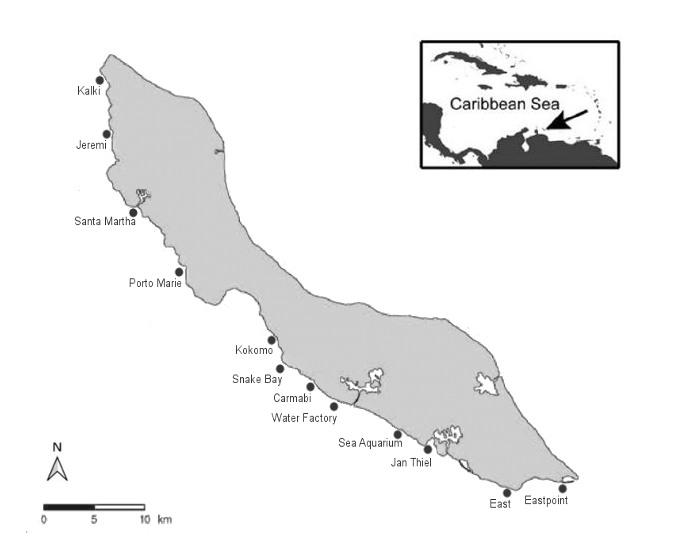


**Figure S1.**

Locations of the 12 surveyed coral reef stations on Curaçao in the Southern Caribbean. Coordinates are provided in Online resource 11.

**
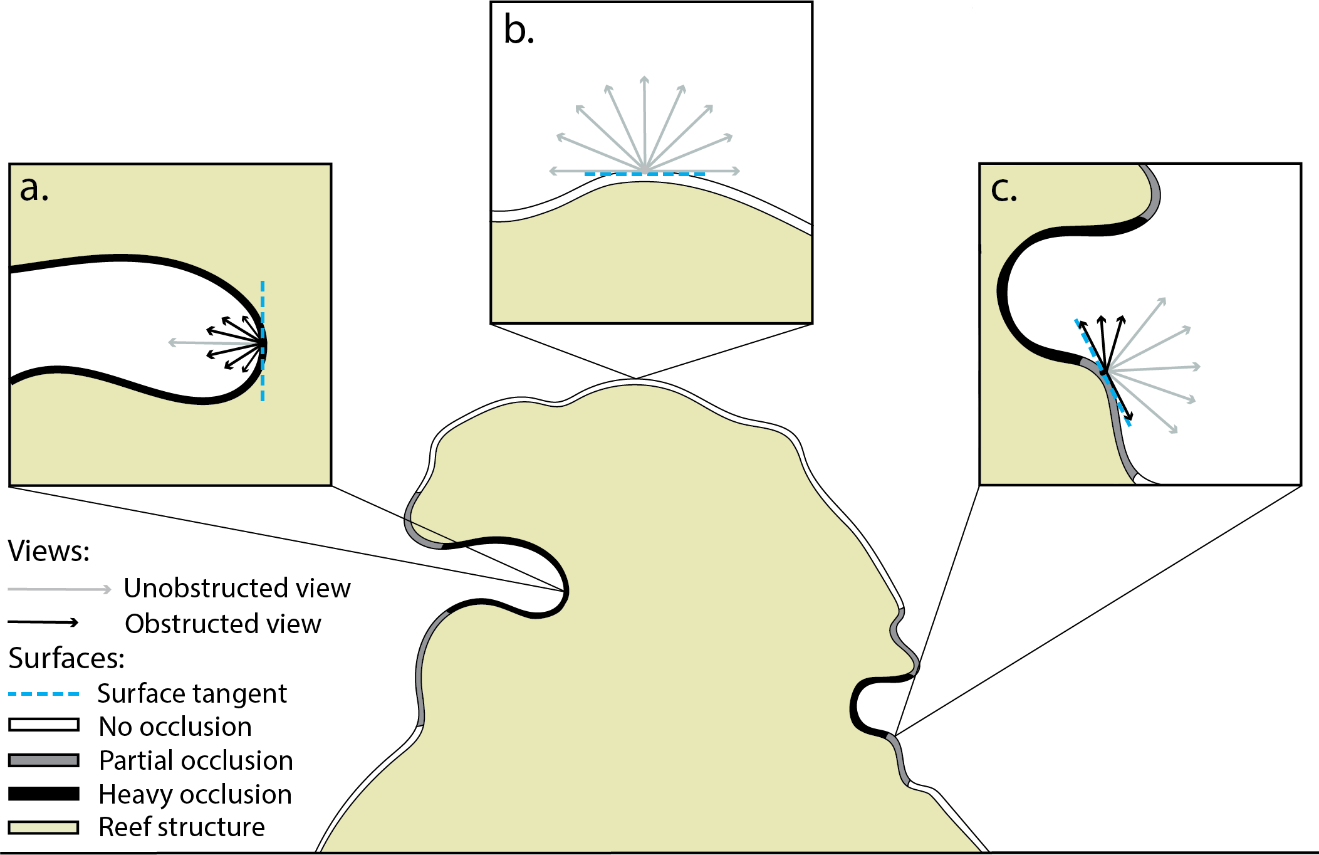
**

**Figure S2.**

Illustration of reef structure surface classification using an Ambient Occlusion algorithm. For each element of the modeled surface, the exposure index is computed as the normalized sum of the unobstructed views, divided by the total number of views (*n* = 256 views per surface element). As a result, cryptic surfaces (as in surface a) will exhibit a lower exposure index than those surfaces completely exposed or with low occlusion (e.g. surfaces b & c).

**Figure S3.**

1500

1000

500

0

Frequency

Exposure index

0.0

0.2

0.4

0.6

0.8

1.0

Frequency distributions of exposure indices for the calibration of cryptic and exposed surface annotation. Typical exposure indices of cryptic surfaces (dark gray) were obtained by applying the Ambient Occlusion algorithm to 3D models of cavity surfaces (*n* = 3). Indices of exposed reef surfaces (light gray) were obtained from 3D models of flat reef tops (*n* = 3). Each model consists of tens of thousands of individual surface elements that each return an exposure index. Values towards 0 mean that the modeled surface is predominantly shaded by other reef surfaces (*see* Fig. S2a), while values towards 1 mean that the surface element is largely exposed, that is, in direct line of site to an external observer above or next to the reef (see Fig. S2b, c). The intersection point between the two distributions (17.5 %, white arrow) was used as a threshold to automatically annotate all surface elements in our 191 reef reconstructions to either cryptic or exposed substrate.


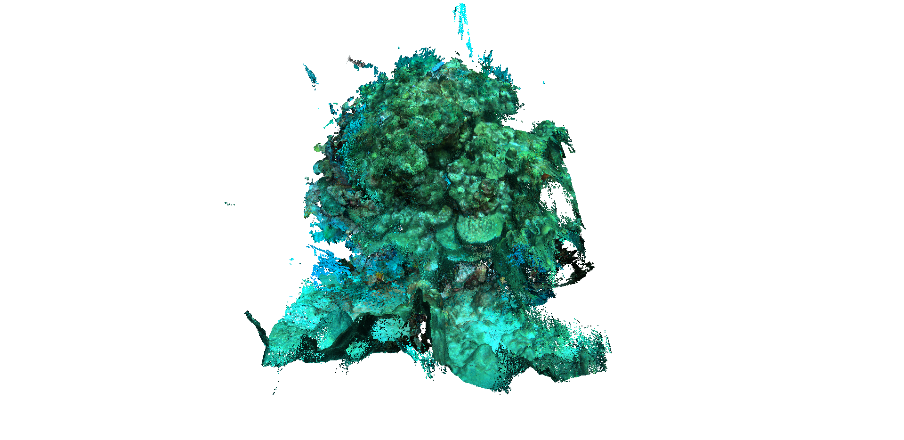

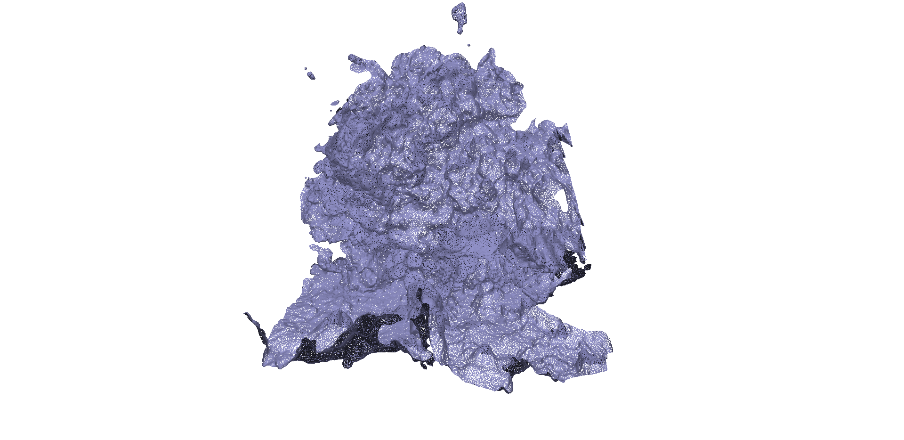

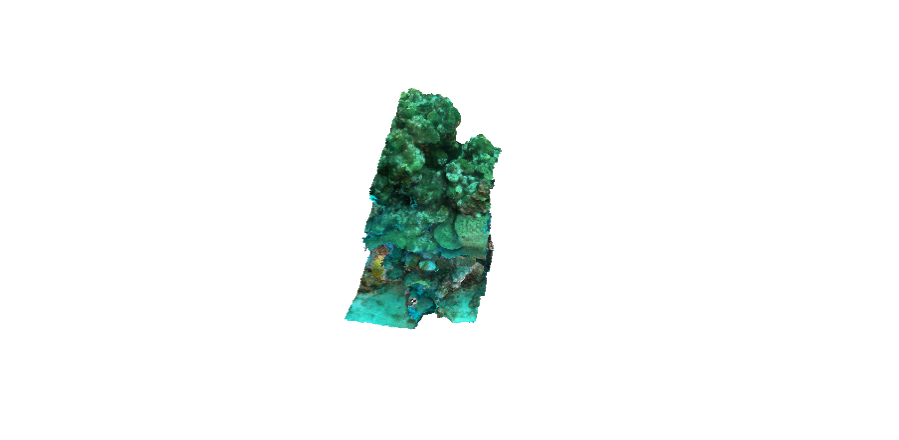

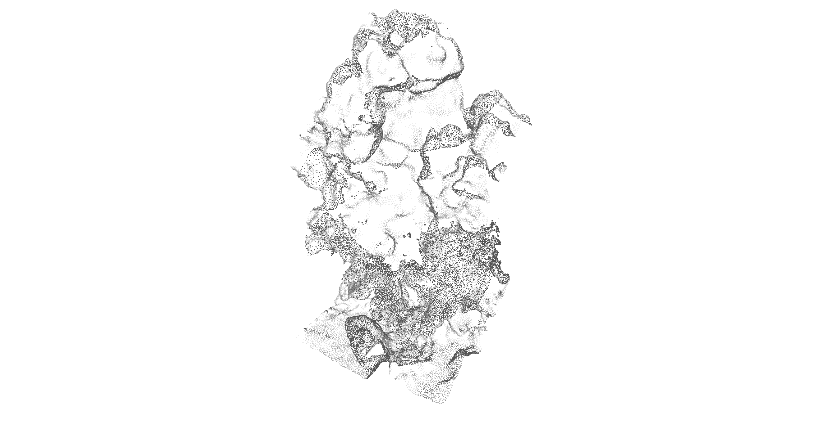

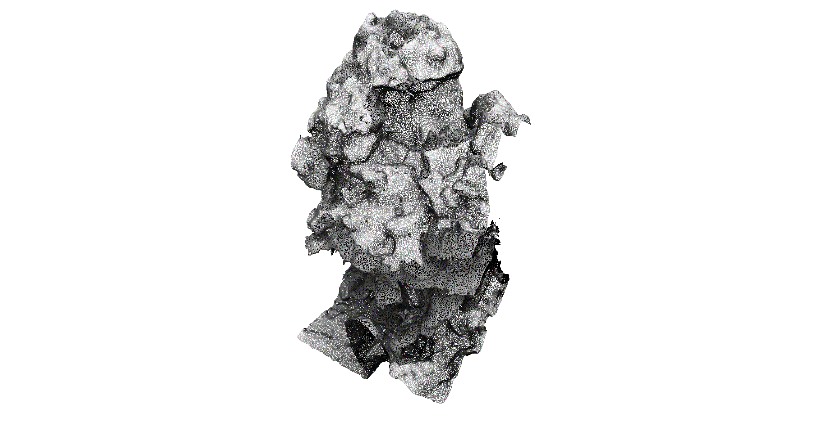

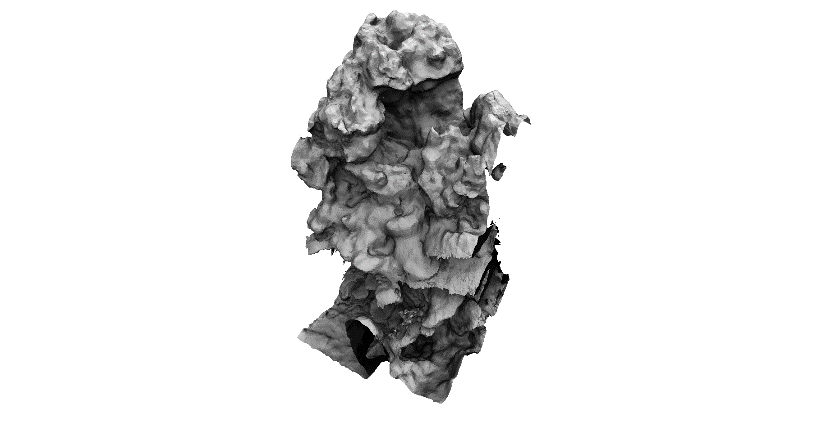


**A**

**B**

**C**

**D**

**E**

**F**

**Figure S4.**

Processing steps of structure-from-motion based 3D reconstructions: (A) high density 3D point cloud, (B) untextured surface mesh obtained from the point cloud and (C) fully textured 3D model of the sample quadrat cropped to 1m x 1m. Light exposure was estimated by (D) resampling the high-density point cloud to 5 mm, (E) smoothing the untextured surface with a two-step Gaussian filter and (F) applying the ambient occlusion algorithm. Note that cryptic surfaces appear darker than exposed surfaces.

*Lobophora spp.*

*Dictyota spp.*

**A**

**B**

**C**

**D**

**E**

**F**

**Figure S5.**

Relationships of algal canopy height and area-normalized tissue volume (A, B, n = 9, error bars are SE), area-normalized ash-free dry weight (AFDW, C, D, n = 3), and area-normalized organic carbon weight (E, F, n = 3) for Dictyota spp. (A, C, E) and Lobophora spp. (B, D, F, *see* Tables S2 and S4 for underlying data).

Benthic cyanobacteria

Turf algae

**A**

**B**

**C**

**D**

**Figure S6.**

Relationship of algal canopy height with area-normalized ash-free dry weight (AFDW, A, B), and organic carbon weight (C, D) for turf algae (A, C, n = 6) and benthic cyanobacterial mats (B, D, n = 8).

**Figure S7.**

Proportions (mean ± SE) of organic carbon (black bars), and organic nitrogen (grey bars), as well as absolute organic tissue (i.e. ash-free dry weight, whole bars) for erect reef organisms (i.e. massive sponges and gorgonians). n = 3 – 4 (*see* Table S1 for individual sample sizes). Raw data on biomass and elemental composition are provided in Online resource 2.

**Figure S8.**

Proportions (mean ± SE) of organic carbon (black bars), and organic nitrogen (grey bars), as well as absolute organic tissue (i.e. ash-free dry weight, whole bars) for non-erect reef organisms. n = 3 – 9 (*see* Table S2 for individual sample sizes). Corals were included in this category based on their morphological characteristics (i.e. thin layer of organic tissue on underlying inorganic skeleton). Raw data on biomass and elemental composition are provided in Online resource 2.

p = 3.68E-09

R^2^ = 0.16

p = 0.008

R^2^ = 0.03

p = 2.76E-08

R^2^ = 0.15

**A**

**B**

**C**

**D**

**E**

**F**

**G**


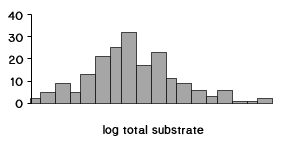


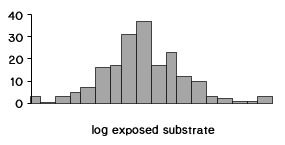


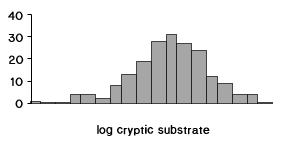


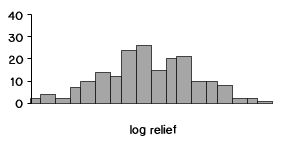


Figure S9.

Histogram plots and linear regressions of total (a, b), exposed (c, d), and cryptic (e, f) substrate areas (in m^2^ substrate area per m^2^ projected reef) using relief as predictor variable (g). All data were log transformed to eliminate positive skew (a, c, e, g) and the resulting least squares predictions (black lines) were back-transformed before plotting (b, d, f). *n* = 191.

**Table S1.** Volume-normalized biomass (i.e. biomass per unit tissue volume, mean ± SE) of erect reef organisms (i.e. gorgonians and massive sponges) in terms of ash-free dry weight and organic carbon in g per cm^3^.

| Organism | Ash-free dry weight  [g cm^-3^] | Organic carbon  [g cm^-3^] | n |
| --- | --- | --- | --- |
| **Gorgonians** | 0.113 ± 0.032 | 0.051 ± 0.015 | 14 |
| *Eunicea sp* | 0.039 ± 0.005 | 0.014 ± 0.001 | 3 |
| *Gorgonia flabellum* | 0.286 ± 0.033 | 0.135 ± 0.014 | 4 |
| *Plexaura sp* | 0.039 ± 0.003 | 0.015 ± 4.8E-04 | 4 |
| *Pseudoplexaura porosa* | 0.055 ± 0.009 | 0.024 ± 0.004 | 3 |
| **Massive sponges** | 0.080 ± 0.006 | 0.036 ± 0.003 | 57 |
| *Agelas clathrodes* | 0.076 ± 0.003 | 0.034 ± 0.003 | 3 |
| *Agelas conifera* | 0.097 ± 0.022 | 0.045 ± 0.010 | 3 |
| *Agelas sventres* | 0.077 ± 0.005 | 0.034 ± 0.002 | 3 |
| *Aiolochroia crassa* | 0.225 ± 0.013 | 0.104 ± 0.006 | 3 |
| *Aplysina archeri* | 0.106 ± 0.015 | 0.050 ± 0.006 | 4 |
| *Aplysina cauliformis* | 0.127 ± 0.006 | 0.059 ± 0.003 | 3 |
| *Aplysina lacunosa* | 0.117 ± 0.022 | 0.052 ± 0.010 | 3 |
| *Biemna sp* | 0.065 ± 0.011 | 0.025 ± 0.004 | 3 |
| *Callyspongia plicifera* | 0.028 ± 0.002 | 0.012 ± 0.001 | 4 |
| *Callyspongia vaginalis* | 0.037 ± 0.005 | 0.018 ± 0.002 | 3 |
| *Desmapsamma anchorata* | 0.044 ± 0.002 | 0.019 ± 0.001 | 3 |
| *Ectyoplasia ferox* | 0.093 ± 0.004 | 0.043 ± 0.002 | 3 |
| *Ircinia campana* | 0.098 ± 0.016 | 0.043 ± 0.006 | 4 |
| *Ircinia felix* | 0.071 ± 0.004 | 0.027 ± 0.002 | 3 |
| *Ircinia strobilina* | 0.055 ± 0.006 | 0.022 ± 3.2E-04 | 3 |
| *Neofibularia nolitangere* | 0.051 ± 0.005 | 0.021 ± 0.002 | 3 |
| *Niphates erecta* | 0.046 ± 0.011 | 0.020 ± 0.005 | 3 |
| *Xestospongia muta* | 0.037 ± 0.004 | 0.015 ± 0.002 | 3 |

**Table S2:** Area-normalized biomass (i.e. biomass per unit tissue surface area, mean ± SE) of all sampled organisms (except gorgonians and massive sponges) in terms of ash-free dry weight and organic carbon in g per cm^2^. Superscripts on coral species indicate coral classification as massive corals (*), branching corals (^+^), encrusting corals (^@^), foliose corals (^&^), sheeting corals (^$^), stalking corals (^^), and solitary corals (^).

| Organism | Ash-free dry weight  [g cm^-2^] | Organic carbon  [g cm^-2^] | n |
| --- | --- | --- | --- |
| **Scleractinian corals** | 0.061 ± 0.007 | 0.018 ± 0.003 | 43 |
| *Acropora cervicornis*^+^ | 0.017 ± 0.003 | 0.004 ± 3.9E-04 | 4 |
| *Agaricia agaricites (sheeting)*^@, $^ | 0.096 ± 0.015 | 0.030 ± 0.008 | 3 |
| *Agaricia agaricites (foliose)*^&^ | 0.015 ± 0.002 | 0.002 ± 2.7E-04 | 3 |
| *Eusmilia fastigiata*^^ | 0.066 ± 0.016 | 0.019 ± 0.006 | 3 |
| *Madracis mirabilis*^+,^ ^^ | 0.099 ± 0.012 | 0.014 ± 0.002 | 3 |
| *Montastrea cavernosa** | 0.087 ± 0.014 | 0.021 ± 0.003 | 3 |
| *Mycetophyllia sp*^@, $^ | 0.052 ± 0.008 | 0.016 ± 0.003 | 3 |
| *Orbicella annularis** | 0.063 ± 0.005 | 0.031 ± 0.015 | 3 |
| *Orbicella faveolata** | 0.138 ± 0.019 | 0.037 ± 0.012 | 3 |
| *Porites astreoides*^@^ | 0.014 ± 0.003 | 0.003 ± 4.7E-04 | 3 |
| *Porites porites*^+^ | 0.015 ± 0.005 | 0.005 ± 0.001 | 3 |
| *Pseudodiploria strigosa** | 0.136 ± 0.012 | 0.050 ± 0.008 | 3 |
| *Siderastrea sidereal** | 0.010 ± 0.002 | 0.002 ± 0.001 | 3 |
| *Tubastrea coccinea*^ | 0.055 ± 0.006 | 0.024 ± 0.004 | 3 |
| **Encrusting sponges** | 0.042 ± 0.013 | 0.019 ± 0.006 | 21 |
| *Clathria sp* | 0.006 ± 0.001 | 0.003 ± 4.6E-04 | 4 |
| *Halisarca caerulea* | 0.007 ± 0.001 | 0.003 ± 4.1E-04 | 4 |
| *Monanchora arbuscula* | 0.007 ± 2.8E-04 | 0.003 ± 2.1E-04 | 3 |
| *Phorbas amaranthus* | 0.026 ± 0.005 | 0.013 ± 0.002 | 3 |
| *Plakortis sp* | 0.154 ± 0.007 | 0.071 ± 0.003 | 4 |
| *Scopalina ruetzleri* | 0.040 ± 0.007 | 0.017 ± 0.003 | 3 |
| **Non-calcifying phototrophs** | 0.003 ± 1.1E-04 | 0.001 ± 5.2E-05 | 20 |
| Benthic cyanobacteria | 0.00038 ± 3.9E-05 | 1.6E-04 ± 1.5E-05 | 8 |
| *Dictyota spp* | 0.003 ± 7.8E-05 | 0.001 ± 2.5E-05 | 3 |
| *Lobophora spp* | 0.007 ± 5.7E-05 | 0.003 ± 3.9E-05 | 3 |
| Turf algae | 0.001 ± 2.6E-05 | 0.001 ± 1.7E-05 | 6 |
| **Calcifying algae** | 0.012 ± 0.002 | 0.003 ± 0.001 | 10 |
| Crustose coralline algae | 0.013 ± 0.002 | 0.002 ± 2.6E-04 | 4 |
| *Halimeda sp* | 0.006 ± 0.001 | 0.002 ± 2.3E-04 | 3 |
| *Peyssonnellia spp* | 0.016 ± 0.003 | 0.006 ± 0.001 | 3 |
| **Other** | 0.018 ± 0.004 | 0.008 ± 0.002 | 15 |
| *Didemnum sp* | 0.004 ± 1.7E-04 | 0.001 ± 2.4E-04 | 3 |
| *Hydrozoa spp* | 0.014 ± 0.005 | 0.008 ± 0.003 | 5 |
| *Lithophaga sp* | 0.014 ± 0.001 | 0.003 ± 2.0E-04 | 3 |
| *Spirobranchus giganteus* | 0.036 ± 0.005 | 0.018 ± 0.002 | 4 |

Table S3.

Illustration of geometrical approximations of massive sponge tissue volumes (similarly applied to gorgonians). Empty spaces of large oscula were also estimated and subtracted from total volume.

| Description | Equation | Geometrical shape | | Example |
| --- | --- | --- | --- | --- |
| Cylinder | $V= \pi r^{2}h$ | ***r***  ***h*** | 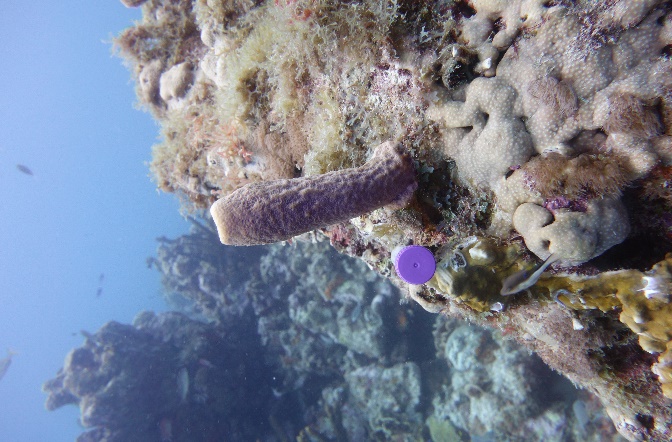 | |
| Rectangular prism | $V=l w h$ | *l*  ***h***  ***w*** | 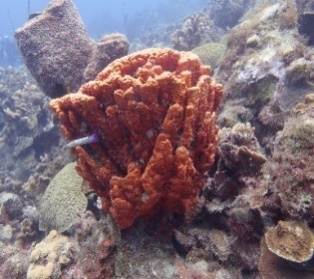 | |
| Cone | $V= \pi r^{2}\frac{h}{3}$ | *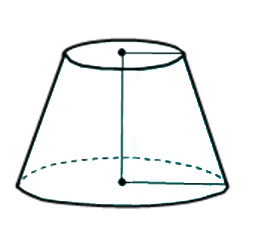*  ***r***  ***r_1_***  ***r_2_***  ***h***  ***h*** | 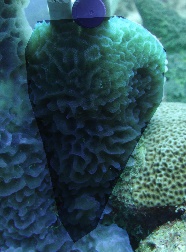 | |
| Truncated cone | $V= \frac{1}{3}\pi\left( r_{1}^{2}+r_{1}r_{2}+r_{2}^{2} \right)h$ |  | 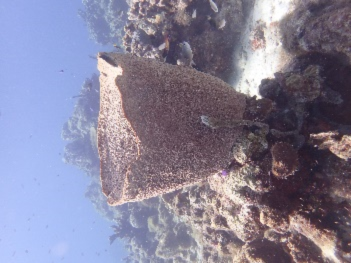 | |
| Ellipsoid | $V=\frac{4}{3} \pi a b c$ | ***b***  ***c***  ***a*** | 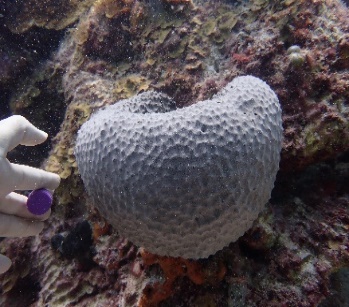 | |

**Table S4.** Dimensions of algal tissue samples for estimating the relationship between canopy height and area-normalized biovolume. Canopy height for each sample was measured three times randomly across the sampled area and then averaged. Linear regression was performed on data grouped by canopy height (Fig. S4a,b).

| Sample | Planar area  [m^2^] | Canopy height  [m] | Tissue surface area  [m^2^] | Tissue volume [m^3^] | Biovolume  [m^3^ m^-2^] |
| --- | --- | --- | --- | --- | --- |
| Dictyota spp. | 0.008 | 0.004 | 0.007 | 2.9E-07 | 3.6E-05 |
| Dictyota spp. | 0.003 | 0.004 | 0.002 | 9.0E-08 | 3.6E-05 |
| Dictyota spp. | 0.003 | 0.005 | 0.003 | 1.1E-07 | 4.3E-05 |
| Dictyota spp. | 0.007 | 0.014 | 0.012 | 4.7E-07 | 6.5E-05 |
| Dictyota spp. | 0.003 | 0.038 | 0.004 | 1.8E-07 | 7.1E-05 |
| Dictyota spp. | 0.003 | 0.034 | 0.005 | 2.2E-07 | 8.7E-05 |
| Dictyota spp. | 0.008 | 0.052 | 0.025 | 9.9E-07 | 1.2E-04 |
| Dictyota spp. | 0.003 | 0.091 | 0.009 | 3.8E-07 | 1.5E-04 |
| Dictyota spp. | 0.003 | 0.083 | 0.010 | 4.1E-07 | 1.6E-04 |
| Lobophora spp. | 0.006 | 0.005 | 0.011 | 7.7E-07 | 1.2E-04 |
| Lobophora spp. | 0.003 | 0.003 | 0.005 | 3.6E-07 | 1.4E-04 |
| Lobophora spp. | 0.003 | 0.003 | 0.005 | 3.7E-07 | 1.5E-04 |
| Lobophora spp. | 0.016 | 0.010 | 0.028 | 2.0E-06 | 1.2E-04 |
| Lobophora spp. | 0.003 | 0.009 | 0.008 | 5.3E-07 | 2.1E-04 |
| Lobophora spp. | 0.003 | 0.010 | 0.007 | 4.8E-07 | 1.9E-04 |
| Lobophora spp. | 0.010 | 0.018 | 0.016 | 1.1E-06 | 1.1E-04 |
| Lobophora spp. | 0.003 | 0.021 | 0.007 | 4.9E-07 | 1.9E-04 |
| Lobophora spp. | 0.003 | 0.018 | 0.008 | 5.3E-07 | 2.1E-04 |

**Supplementary text 1.** Simulating direct light to discern exposed and cryptic reef surfaces

Cryptic habitats are broadly defined in the literature, ranging from large caves, to crevices and holes, to the spaces between sand grains (Choi and Ginsburg 1983; Ginsburg 1983). We defined exposed and cryptic surfaces solely based on their orientation, whereby exposed surfaces are directly visible for a human observer swimming over the reef whereas cryptic surfaces are out of direct view because they are hidden behind exposed surfaces. We aimed at automating the classification of the 3D reconstructed surfaces into either *exposed* or *cryptic* according to the above criteria. For this purpose, we employed the Ambient Occlusion (Landis 2002) algorithm which is a 3D object shading and rendering technique in computer graphics. It estimates the percentage of occlusion of direct light by nearby objects according to:

$AO(x, \vec{n}=\frac{1}{\pi}\cdot\int_{\vec{\omega}\in\Omega}^{0} V\left( x, \vec{\omega} \right)\left( \vec{n}\cdot\vec{\omega} \right)d\hat{\omega}$ (1)

where $\vec{n}$ is the normal vector of the surface tangent and V is the visibility function along the direction of $\vec{\omega}$. The function returns the value 0 if the surface is fully occluded, and 1 if the surface is fully exposed. We used the Meshlab implementation of the AO algorithm (Sabbadin et al. 2016) with 128 simulated 60-degree conical sources and a directional bias of 0.5. To calibrate the annotation into exposed and cryptic surfaces, we first applied the AO algorithm on selected 3D reconstructions of exposed reef tops and cryptic overhangs, which yielded the brighter and darker populations of exposure indices, respectively, in Fig. S3. The exposure threshold for automatic annotations was selected where the two frequency histograms meet. This threshold provides the maximum likelihood to correctly annotate surface elements of unknown type for an expected bimodal distribution. While this approach can correctly annotate digital surfaces based on their orientation and the presence of surfaces in their surroundings, our definition of cryptic habitats is limited and would benefit from ecological assessments of how light mediates the spatial extent of exposed and cryptic reef communities.

**Supplementary text 2.** Equations used to calculate 3D surface area, biovolume, and biomass of benthic coral reef organisms.

3D surface area (SA) of benthic group *i* on horizontal (*ho*), vertical (*ve*), and cryptic (*cr*) substrates, with A as substrate surface area determined by 3D reconstructions (see methods) and C as relative cover, was calculated by:

$\mathrm{SA}_{ho, i}=A_{ho, i}\cdot C_{ho. i}$ (2)

$\mathrm{SA}_{ve, i}=A_{ve, i}\cdot C_{ve, i}$ (3)

$\mathrm{SA}_{cr, i}=A_{cr, i}\cdot C_{cr, i}$ (4)

Uncertainty ($\delta)$ for 3D surface area on individual substrates (shown here for cryptic substrates) was propagated according to:

${\delta SA}_{\mathrm{cr}, i}=\mathrm{SA}_{cr, i}\cdot\sqrt{\left( \frac{{\delta A}_{cr, i}}{A_{cr, i}} \right)^{2}+\left( \frac{{\delta C}_{cr, i}}{C_{cr, i}} \right)^{2}}$ (5)

Total 3D surface area (TSA) of benthic group *i* and its uncertainty were calculated by:

$\mathrm{TSA}_{i}=\mathrm{SA}_{ho, i}+\mathrm{SA}_{ve, i}+\mathrm{SA}_{cr, i}$ (6)

${\delta TSA}_{i}=\sqrt{\left( {\delta SA}_{ho, i} \right)^{2}+\left( {\delta SA}_{ve, i} \right)^{2}+\left( {\delta SA}_{cr, i} \right)^{2}}$ (7)

Exposed 3D surface area (TSA_E_) of benthic group *i* and its uncertainty were calculated by:

$\mathrm{TSA}_{E, i}=\mathrm{SA}_{ho, i}+\mathrm{SA}_{ve, i}$ (8)

${\delta TSA}_{E, i}=\sqrt{\left( {\delta SA}_{ho, i} \right)^{2}+\left( {\delta SA}_{ve, i} \right)^{2}}$ (9)

Volume of organic tissue (V) for non-erect, roughly isometric organisms (including scleractinian corals) on individual substrates (shown here for horizontal surfaces) and its uncertainty, where d is the tissue thickness, were calculated by:

$V_{ho, i}=\mathrm{TSA}_{ho, i}\cdot d_{ho, i}$ (10)

${\delta V}_{ho, i}=V_{ho, i}\cdot\sqrt{\left( \frac{{\delta TSA}_{ho, i}}{\mathrm{TS}A_{ho, i}} \right)^{2}+\left( \frac{{\delta d}_{ho, i}}{d_{ho, i}} \right)^{2}}$ (11)

Volume for macroalgae (denoted by *M*) on individual substrates (shown here for horizontal surfaces) and its uncertainty, with *D* denoting *Dictyota spp.* and *L* denoting *Lobophora spp.*, while vCH_D_ and vCH_L_ represent the functions of area-normalized tissue volume by canopy height (CH) for Dictyota (Fig. S5a) and Lobophora (Fig. S5b), respectively, were calculated by:

$V_{ho, M}=\mathrm{TSA}_{ho, M}\cdot\frac{\mathrm{vCH}_{D}(\mathrm{CH}_{ho, D})+\mathrm{vCH}_{L}(\mathrm{CH}_{ho, L})}{2}$ (12)

${\delta V}_{ho, M}=V_{ho, M}\cdot\sqrt{\left( \frac{{\delta TSA}_{ho, M}}{T\mathrm{SA}_{ho, M}} \right)^{2}+\left( {\delta vCH}_{ho, M} \right)^{2}}$ (13)

where ${\delta vCH}_{ho, m}$ is obtained according to:

${\delta vCH}_{ho, M}=\frac{{(vCH}_{D}\left( \mathrm{CH}_{ho, M}+\delta\mathrm{CH}_{ho, M} \right)-\mathrm{vCH}_{D}\left( \mathrm{CH}_{ho, M} \right))+{(vCH}_{L}\left( \mathrm{CH}_{ho, M}+{\delta CH}_{ho, M} \right)-\mathrm{vCH}_{L}\left( \mathrm{CH}_{ho, M} \right))}{\mathrm{vCH}_{D}\left( \mathrm{CH}_{ho, M} \right)+\mathrm{vCH}_{L}\left( \mathrm{CH}_{ho, M} \right)}$ (14)

Total volume of benthic group *i* and its uncertainty were calculated by:

$V_{i}=V_{ho, i}+V_{ve, i}+V_{cr, i}$ (15)

${\delta V}_{i}=\sqrt{\left( {\delta V}_{ho, i} \right)^{2}+\left( {\delta V}_{ve, i} \right)^{2}+\left( {\delta V}_{cr, i} \right)^{2}}$ (16)

Exposed volume (V_E_) of benthic group *i* and its uncertainty were calculated by:

$V_{E, i}=V_{ho, i}+V_{ve, i}$ (17)

${\delta V}_{E, i}=\sqrt{\left( {\delta V}_{ho, i} \right)^{2}+\left( {\delta V}_{ve, i} \right)^{2}}$ (18)

Biomass of organic tissue (B) for erect organisms (gorgonians and massive sponges) on individual substrates (shown here for vertical surfaces) and its uncertainty, where mv is the biomass normalized to tissue volume, were calculated by:

$B_{\mathrm{ve}, i}=V_{ve, i}\cdot\mathrm{mv}_{\mathrm{ve}, i}$ (19)

${\delta B}_{\mathrm{ve}, i}=V_{ve, i}\cdot\sqrt{\left( \frac{{\delta V}_{ve, i}}{V_{ve, i}} \right)^{2}+\left( \frac{{\delta mv}_{ve, i}}{\mathrm{mv}_{ve, i}} \right)^{2}}$ (20)

Biomass of organic tissue (B) for non-erect, roughly isometric organisms (including scleractinian corals) on individual substrates (shown here for horizontal surfaces) and its uncertainty, where ms is the biomass normalized to surface area, were calculated by:

$B_{ho, i}=\mathrm{TSA}_{ho, i}\cdot\mathrm{ms}_{ho, i}$ (21)

${\delta B}_{ho, i}=B_{ho, i}\cdot\sqrt{\left( \frac{{\delta TSA}_{ho, i}}{\mathrm{TS}A_{ho, i}} \right)^{2}+\left( \frac{{\delta ms}_{ho, i}}{\mathrm{ms}_{ho, i}} \right)^{2}}$ (22)

Organic biomass for turf algae or benthic cyanobacteria on individual substrates (shown here for horizontal surfaces) and its uncertainty, with mCH_i_ representing the area-normalized biomass (Fig. S6a,b), were calculated by:

$B_{ho, i}=\mathrm{TSA}_{ho, i}\cdot\mathrm{mCH}_{i}(\mathrm{CH}_{ho, i})$ (23)

${\delta B}_{ho, i}=B_{ho, i}\cdot\sqrt{\left( \frac{{\delta TSA}_{ho, i}}{T\mathrm{SA}_{ho, i}} \right)^{2}+\left( {\delta mCH}_{ho, i} \right)^{2}}$ (24)

where ${\delta mCH}_{ho, i}$ is obtained according to:

${\delta mCH}_{ho, i}=\frac{(\mathrm{mCH}_{i}\left( \mathrm{CH}_{ho, i}+\delta\mathrm{CH}_{ho, i} \right)-\mathrm{mCH}_{i}\left( \mathrm{CH}_{ho, i} \right))}{\mathrm{mCH}_{i}\left( \mathrm{CH}_{ho, i} \right)}$ (25)

Organic biomass for macroalgae on individual substrates (shown here for horizontal surfaces) and its uncertainty, with mCH_D_ and mCH_L_ representing the functions of area-normalized biomass by canopy height for Dictyota (Fig. S5c) and Lobophora (Fig. S5d), respectively, were calculated by:

$B_{ho, M}=\mathrm{TSA}_{ho, M}\cdot\frac{\mathrm{mCH}_{D}(\mathrm{CH}_{ho, M})+\mathrm{mCH}_{L}(\mathrm{CH}_{ho, M})}{2}$ (26)

${\delta B}_{ho, M}=B_{ho, M}\cdot\sqrt{\left( \frac{{\delta TSA}_{ho, M}}{\mathrm{TS}A_{ho, M}} \right)^{2}+\left( {\delta mCH}_{ho, M} \right)^{2}}$ (27)

where ${\delta mCH}_{ho, M}$ is obtained according to:

${\delta mCH}_{ho, M}=\frac{{(mCH}_{D}\left( \mathrm{CH}_{ho, M}+\delta\mathrm{CH}_{ho, M} \right)-\mathrm{mCH}_{D}\left( \mathrm{CH}_{ho, M} \right))+{(mCH}_{L}\left( \mathrm{CH}_{ho, M}+{\delta CH}_{ho, M} \right)-\mathrm{mCH}_{L}\left( \mathrm{CH}_{ho, M} \right))}{\mathrm{mCH}_{D}\left( \mathrm{CH}_{ho, M} \right)+\mathrm{mCH}_{L}\left( \mathrm{CH}_{ho, M} \right)}$ (28)

Total organic biomass of benthic group *i* and its uncertainty were calculated by:

$B_{i}=B_{ho, i}+B_{ve, i}+B_{cr, i}$ (29)

${\delta B}_{i}=\sqrt{\left( {\delta B}_{ho, i} \right)^{2}+\left( {\delta B}_{ve, i} \right)^{2}+\left( {\delta B}_{cr, i} \right)^{2}}$ (30)

Exposed organic biomass (B_E_) of benthic group *i* and its uncertainty were calculated by:

$B_{E, i}=B_{ho, i}+B_{ve, i}$ (31)

${\delta B}_{E, i}=\sqrt{\left( {\delta B}_{ho, i} \right)^{2}+\left( {\delta B}_{ve, i} \right)^{2}}$ (32)

Organic carbon stocks and associated uncertainties were calculated using equations 19 – 32, while replacing area- and volume-normalized organic biomass with area- and volume-normalized organic carbon mass (Tables S1, S2). For non-calcifying phototrophs, the relationships mCH_i_ were also replaced with the functions describing area-normalized organic carbon mass by canopy height (Fig. S5e,f for *Dictyota spp.* and *Lobophora spp.*, Fig. S6c, d for turf algae and benthic cyanobacteria).

**ADDITIONAL ONLINE RESOURCES**

**Online resource 2.** Volume- and biomass conversions. ‘Samples’ lists raw results of the tissue analyses. Organisms are summarized in ‘summary’ and different benthic groups are summarized in ‘communities’. Volume- and biomass standardizations for fleshy algae and subgroups thereof are based on their average canopy heights.

**Online resource 3.** Hidden cave surfaces and volumes. All surveyed quadrats are listed in rows, and columns show individual (hardly accessible and, thus, hand-measured) cavities in each quadrat. ‘hidden cavity surface areas’ displays total surface areas and ‘hidden cavity volumes’ displays cavity volumes, as approximated from simple geometrical shapes.

**Online resource 4.** Coral cryptic surface ratios. Proportion of cryptic surface in percent of live coral surface for individuals of sheeting and stalking corals.

**Online resource 5.** Percent cover. ‘horizontal percent cover’ sheet lists raw results from horizontal (i.e. top-down) coral point counts. Sites are summarized in ‘horizontal site summaries’ and communities are summarized in ‘horizontal communities’. The tab ‘horizontal cover incl layers’ displays cover data that was used to calculate 3D metrics of reef communities (*see* Methods – ‘Relative cover on exposed and cryptic reef surfaces’). Percent cover and respective site summaries for percent cover on vertical and cryptic surfaces are provided in the following tabs.

**Online resource 6.** Absolute 3D surface cover. Surface areas of individual benthic biota for the total reef (reef summaries), as well as exposed areas (exposed summaries) and cryptic areas (cryptic summaries) individually. Communities are summarized in ‘communities’.

**Online resource 7.** Biovolumes and canopy heights. Canopy height measurements for individual surface types (horizontal, vertical, and cryptic) are listed in ‘canopy heights’, and summarized by site and surface type in ‘canopy summaries’. ‘emergent organisms’ lists *in situ* measured biovolumes of massive sponges and gorgonians. Total volumes are summarized by site in ‘reef volumes’ (total reef), ‘exposed volumes’ (exposed reef), and ‘cryptic volumes’ (cryptic reef). Benthic groups are summarized in ‘communities’.

**Online resource 8.** Standing stock of ash-free dry weights. Total weights are summarized by site in ‘reef summaries’ (total reef), ‘exposed summaries’ (exposed reef), and ‘cryptic summaries’ (cryptic reef). Benthic groups are summarized in ‘communities’.

**Online resource 9.** Standing stock of organic carbon. Total weights are summarized by site in ‘reef summaries’ (total reef), ‘exposed summaries’ (exposed reef), and ‘cryptic summaries’ (cryptic reef). Benthic groups are summarized in ‘communities’.

**Online resource 10.** Standing stock of organic nitrogen. Total weights are summarized by site in ‘reef summaries’ (total reef), ‘exposed summaries’ (exposed reef), and ‘cryptic summaries’ (cryptic reef). Benthic groups are summarized in ‘communities’.

**Online resource 11.** Site coordinates and substrate surface areas. ‘substrates’ sheet lists relief and surface areas of horizontal, vertical, and cryptic reef surfaces, as well as all surfaces combined (i.e. total surface area). Sites are summarized and coordinates are provided in ‘site summaries’.

**Coral reef 3D reconstructions.** 3D models of 191 m^2^ of coral reef benthos are provided in an interactive format on Sketchfab ([www.sketchfab.com](http://www.sketchfab.com)) under the account ‘coralreefs.kornder’. Models are named by site and quadrat (e.g. Carmabi 1 – 16 chronologically relate to the 16 rows with site = Carmabi across all Online resources).

**References**

Choi DR, Ginsburg RN (1983) Distribution of coelobites (cavity-dwellers) in coral rubble across the Florida Reef Tract. Coral Reefs 2:165-172

Ginsburg RN (1983) Geological and biological roles of cavities in coral reefs. In: D Barnes (ed). Perspectives on Coral Reefs:143-153

Landis H (2002) Production-ready global illumination. Siggraph course notes 16:11

Sabbadin M, Palma G, Cignoni P, Scopigno R (2016) Multi-view ambient occlusion for enhancing visualization of raw scanning data. Proceedings of the 14th Eurographics Workshop on Graphics and Cultural Heritage:23-32
